# Particle Swarm Optimization with Reinforcement Learning for the Prediction of CpG Islands in the Human Genome

**DOI:** 10.1371/journal.pone.0021036

**Published:** 2011-06-28

**Authors:** Li-Yeh Chuang, Hsiu-Chen Huang, Ming-Cheng Lin, Cheng-Hong Yang

**Affiliations:** 1 Institute of Biotechnology and Chemical Engineering, I-Shou University, Kaohsiung, Taiwan; 2 Institute of Biomedical Engineering, National Cheng Kung University, Tainan, Taiwan; 3 Department of Physical Medicine and Rehabilitation, Chia-Yi Christian Hospital, Chia-Yi, Taiwan; 4 Department of Electronic Engineering, National Kaohsiung University of Applied Sciences, Kaohsiung, Taiwan; 5 Department of Network Systems, Toko University, Chiayi, Taiwan; Dana-Farber Cancer Institute, United States of America

## Abstract

**Background:**

Regions with abundant GC nucleotides, a high CpG number, and a length greater than 200 bp in a genome are often referred to as CpG islands. These islands are usually located in the 5′ end of genes. Recently, several algorithms for the prediction of CpG islands have been proposed.

**Methodology/Principal Findings:**

We propose here a new method called CPSORL to predict CpG islands, which consists of a complement particle swarm optimization algorithm combined with reinforcement learning to predict CpG islands more reliably. Several CpG island prediction tools equipped with the sliding window technique have been developed previously. However, the quality of the results seems to rely too much on the choices that are made for the window sizes, and thus these methods leave room for improvement.

**Conclusions/Significance:**

Experimental results indicate that CPSORL provides results of a higher sensitivity and a higher correlation coefficient in all selected experimental contigs than the other methods it was compared to (CpGIS, CpGcluster, CpGProd and CpGPlot). A higher number of CpG islands were identified in chromosomes 21 and 22 of the human genome than with the other methods from the literature. CPSORL also achieved the highest coverage rate (3.4%). CPSORL is an application for identifying promoter and TSS regions associated with CpG islands in entire human genomic. When compared to CpGcluster, the islands predicted by CPSORL covered a larger region in the TSS (12.2%) and promoter (26.1%) region. If *Alu sequences* are considered, the islands predicted by CPSORL (*Alu*) covered a larger TSS (40.5%) and promoter (67.8%) region than CpGIS. Furthermore, CPSORL was used to verify that the average methylation density was 5.33% for CpG islands in the entire human genome.

## Introduction

CpG islands are short sequences that preserve a high concentration of the two nucleic acids Cytosine (C) and Guanine (G). The letter ‘p’ in CpG represents the phosphodiester bonds that appear between the nucleic acids C and G. CpG islands were first identified by Tykocinski and Max as small regions that contain the restriction enzyme *HpaII* in the genome and were thus originally called *HpaII* Tiny Fragment (HTF) islands [1].

A definition of CpG islands was first offered by Gardiner-Garden and Frommer (GGF) in 1987 [2]. The original description included the length of the suspected region, which has to exceed 200 bp, the GC content in that region, which has to be higher than 50%, and the observed/expected (O/E) ratio, which has to surpass a value of 0.6. Since biological experiments have proven that there could be two *Alu* sequences in a CpG island, Takai and Jones revised the GGF criteria of CpG islands in 2002 [3]. Their modified definition requires that the minimum length of the suspected region is 500 bp and that the required GC content and O/E ratio are 55% and 0.65, respectively. The *Alu* endonuclease is so-named because it was first isolated from *Arthrobacter luteus*. *Alu* sequences are highly repetitive short interspersed elements with an approximate consensus sequence of about 280 bp. Some of these sequences have a relative high GC content and O/E ratio [2], [3]. Recently, various algorithms have been adopted in the literature to predict CpG islands, e.g., CpGIS [3], CpGPlot [4], CpGProD [5] and CpGcluster [6], but most of these tools use the sliding window technique with the GC content, O/E ratio and length thresholds as the main parameters; CpGcluster uses the distance between CpG dinculeotides.

PSO is a population-based stochastic optimization technique developed by Kennedy and Eberhart [7]. The main advantage of PSO is that it has the ability to converge fast. The individual memory of the particles in PSO can be used to compare information in a search process. To date, PSO has been successfully applied in many fields, including operon prediction [8] and biomarker selection [9], amongst others.

In this study we propose a new prediction method called CPSORL, which combines complementary particle swarm optimization (CPSO) with the reinforcement learning (RL) method to predict CpG islands in the human genome. Reinforcement learning [10] is applied to extend the shorter CpG islands or even combine neighboring CpG islands if prescribed requirements are met (an example comparison of CpG island predictions with and without a reinforcement learning process is show in Figure S1).

The proposed CPSORL method adopts the GGF criteria (GC content ≧50%, O/E ratio ≧0.6, length ≧200 bp) as guidelines for the search for CpG islands. CPSORL is composed of two major steps. First, the input sequence is cut apart into windows, and then the PSO algorithm is used to search for DNA sequences that are in accordance with the GGF criteria. The PSO mechanism is updated iteratively to search for optimal results and identifies the best performing particles in the swarm population [11]. If the PSO particles fall into a local search pattern, the complementary concept enables them to leave this local region and participate in the global search again. In a second step, the length of the predicted CpG island is extended by RL; islands are combined with neighboring islands until the length definition parameters are met [10], [12]. Experimental results indicate that CPSORL provides results of a higher sensitivity and a higher correlation coefficient in all selected experimental contigs than CpGIS, CpGcluster, CpGProd and CpGPlot.

## Results

### Parameter settings

In PSO, four different parameters need to be set: the population size, the number of iterations, and the *C*_1_ and *C*_2_ constants of the update function. The population size in our study was set to 300 [13], the number of iterations was set to 100, and *C*_1_ and *C*_2_ were set to 2 [11]. The CpGIS parameters were: length set to 200 bp, GC content set to 50%, O/E ratio set to 0.6, and the gap between adjacent islands set to 100 bp (http://cpgislands.usc.edu/). CpGcluster parameters used were: p-value threshold of 1E-5 and distance threshold (percentile) of 50. CpGProd and CpGplot were used directly from the internet (http://pbil.univ-lyon1.fr/software/cpgprod_query.html and http://www.ebi.ac.uk/Tools/emboss/cpgplot/index.html).

### Performance measurement

We used five common criteria to determine the prediction accuracy, namely the sensitivity (*SN*), specificity (*SP*), accuracy (*ACC*), performance coefficient (*PC*) and correlation coefficient (*CC*) [14]. The five criteria are defined in Eqs. (1–5). Through these five evaluation criteria the superiority of an algorithm was determined. The calculation processes are shown in detail in Figure S2.
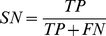
(1)

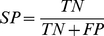
(2)

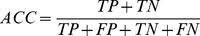
(3)

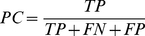
(4)

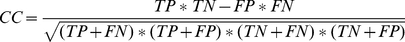
(5)where *TP* is a true positive, *FN* is a false negative, *TN* is a true negative and *FP* is a false positive. We predicted CpG islands under the GGF criteria. Subsequently, we used five evaluation criteria to assess the CpG island prediction performance of all methods.

In addition, the receiver operating characteristic (ROC) curve is introduced to determine equivalence by plotting the fraction of true positives out of the positives (TPR = true positive rate) vs. the fraction of false positives out of the negatives (FPR = false positive rate). Hanson has pointed out that the area under the ROC curve can be used to predict the accuracy of a risk scale [15]. The ROC curve plots the sensitivity against the specificity; the sensitivity and specificity express the accuracy of the CpG island prediction factors.

### Experimental results

We propose an effective hybrid method of CPSO and RL called CPSORL to identify CpG islands in the human genome. In CPSORL, CPSO supplies the updating function to find potential regions of CpG islands, and RL is used to extend and combine CpG islands in order to improve the prediction quality. The CPSO proposed in this study prevents the entrapment of particles in a local optimum. Table 1 shows a comparison of the performance of different methods from the literature for CpG island prediction, such as *SN*, *SP*, *ACC*, *PC*, and *CC*. CPSORL provides *SN*, *PC* and *CC* results that are higher than in other methods it was compared to. We compared CPSORL with various other methods in the literature. Table S1 shows the results in the contig NT_113954.1 for different CpG island prediction tools. Table 2 contains the number of CpG islands located in gene regions identified with CPSORL. A comparison of the number of CpG islands identified in the human genome with different methods is shown in Table 3. Table 4 shows the number of methylation sites identified with CPSORL in chromosomes 21 and 22 of the human genome, and also includes the chromosome length, total length of a CpG island, the number of methylation sites in entire genome, the number of methylation sites selected in CPSORL, and the methylation density of the CpG islands. CPSORL predicted CpG islands with an average methylation density of 5.33% in the entire chromosome; the results are shown in Table 5. Table 6 shows the prediction performance for the entire human chromosome by the proposed method and the methods from the literature.

**Table 1:** Comparison of different methods for CpG island prediction.

*Contig.*	Performance	Methods							
		CpGPlot	CpGcluster	CpGProD	CpGIS	PSO		CPSO	
						without *RL*	with *RL*	without *RL*	with *RL*
**NT_113952.1** **Length = 184355**	SN (%)	56.43	50.46	58.07	83.98	69.22	75.58	77.43	84.88
	SP (%)	100.0	99.95	99.50	99.05	99.61	99.02	99.58	99.05
	ACC (%)	98.09	97.78	97.69	98.39	98.28	97.99	98.61	98.43
	PC (%)	56.42	49.92	52.36	69.59	63.77	62.27	70.91	70.34
	CC (%)	74.38	69.41	68.83	81.25	77.66	75.71	82.49	81.80
**NT_113955.2** **Length = 281920**	SN (%)	47.19	67.15	68.51	85.12	54.47	59.63	77.80	87.38
	SP (%)	100.0	99.72	99.63	99.30	99.96	99.88	99.50	99.61
	ACC (%)	98.08	98.54	98.50	98.79	98.31	98.42	98.71	99.16
	PC (%)	47.14	62.47	62.35	71.78	53.87	57.74	68.67	79.08
	CC (%)	67.94	77.03	76.65	82.96	72.41	74.51	80.85	87.89
**NT_113958.2** **Length = 209483**	SN (%)	51.29	27.16	46.41	82.13	79.27	81.65	81.08	84.11
	SP (%)	99.99	99.94	98.93	98.26	98.13	97.90	98.17	98.34
	ACC (%)	96.90	95.32	95.60	97.24	96.93	96.87	97.08	97.43
	PC (%)	51.24	26.92	40.10	65.36	62.10	62.33	63.80	67.51
	CC (%)	70.38	49.96	56.80	77.63	75.03	75.28	76.41	79.31
**NT_113953.1** **Length = 131056**	SN (%)	22.80	57.32	29.79	74.05	60.20	64.80	70.53	75.65
	SP (%)	100.0	99.74	99.56	98.83	99.27	99.23	99.22	99.13
	ACC (%)	97.76	98.51	97.53	98.11	98.13	98.23	98.38	98.45
	PC (%)	22.80	52.74	25.96	53.23	48.39	51.59	55.91	58.57
	CC (%)	47.21	69.89	43.61	68.64	64.50	67.25	70.90	73.10
**NT_113954.1** **Length = 129889**	SN (%)	31.24	29.86	52.01	76.31	56.92	63.58	70.54	77.68
	SP (%)	100.0	99.46	98.72	97.62	98.40	98.13	98.34	98.23
	ACC (%)	97.47	96.90	97.00	96.83	96.87	96.86	97.32	97.48
	PC (%)	31.24	26.19	38.94	47.05	40.12	42.74	49.22	53.15
	CC (%)	55.17	43.81	54.68	63.29	55.65	58.36	64.72	68.53
**NT_028395.3** **Length = 647850**	SN (%)	27.11	44.89	54.18	76.68	68.97	72.79	72.52	77.02
	SP (%)	100.0	99.47	99.45	98.93	99.27	98.99	9918	98.90
	ACC (%)	97.98	97.53	98.19	98.14	98.19	98.06	98.24	98.12
	PC (%)	27.10	39.26	45.36	59.36	57.49	57.17	59.36	59.25
	CC (%)	51.51	57.21	62.26	73.57	72.21	71.75	73.61	73.48

RL: Reinforcement Learning. SN: Sensitivity. SP: Specificity. ACC: Accuracy. PC: Performance coefficient. CC: Correlation coefficient. Underlined value representing the best results.

**Table 2:** Number of CpG islands located in gene regions identified with CPSORL.

Chr.	Contig	GC% (Average)	CpG island length	CpG island number	Number of genes*
***21***	NT_113952.1	54.34	8,537	12	1(3)
***21***	NT_113955.2	53.04	10,023	15	2(3)
***21***	NT_113958.2	57.01	14,470	19	2(3)
***21***	NT_113953.1	50.92	3,998	8	1(1)
***21***	NT_113954.1	54.53	6,174	10	1(1)
***22***	NT_028395.3	55.40	24,649	38	10(15)

(*)True number of genes in the contig is given in parentheses.

**Table 3:** Comparison of the number of CpG islands identified in the human genome with different methods. *(NCBI.36)*.

	*Chromosome 21*						
	*CpGPlot*	*CpGcluster*	*CpGProD*	*CpGIS*	*PSORL*	*CPSORL*	*CPSORL* *(ALU)*
Chromosome Length (bp)	46,944,329						
Total length of CpG islands	347,334	639,161	1,072,192	1,280,505	1,564,596	1,607,472	926,178
Number of islands predicted	973	2,703	1,091	3,704	2,648	2,813	850
Island coverage (%)^a^	0.73	1.36	2.28	2.73	3.3	3.4	1.97
Island length (bp)							
Average	357	237	983	346	591	571	1,089
Minimum	101	8	500	200	202	202	500
Maximum	3,047	3,028	6,732	1,948	4,020	4,035	4,035
GC-content ± SD (%)	62.17±0.07	65.49±0.07	54.49±0.06	57.98±0.04	53.73±0.05	53.72±0.05	55.60±0.05
CpG island O/E ratio ±SD	0.84±0.1	0.87±0.3	0.63±0.1	0.68±0.1	0.64±0.08	0.65±0.08	0.65±0.09

	*Chromosome 22*						
	*CpGPlot*	*CpGcluster*	*CpGProD*	*CpGIS*	*PSORL*	*CPSORL*	*CPSORL* *(ALU)*
Chromosome Length (bp)	49,691,432						
Total length of CpG islands	679,803	522,748	2,067,653	2,842,255	2,802,675	2,907,983	1,795,617
Number of islands predicted	1,642	2,186	1,903	6,875	4,571	4,882	1,569
Island coverage (%)	1.36	1.05	4.16	5.71	5.64	5.85	3.6
Island length (bp)							
Average	414	239	1,087	413	613	596	1,144
Minimum	200	8	500	200	198	202	500
Maximum	7,902	7,774	8,363	3,339	4,076	4,076	4,076
GC-content ± SD (%)	63.70±0.08	70.23±0.08	55.84±0.07	55.12±0.06	54.50±0.07	54.46±0.07	56.53±0.06
CpG island O/E ratio ±SD	0.84±0.1	0.95±0.3	0.62±0.1	0.68±0.1	0.63±0.05	0.63±0.05	0.64±0.08

SD is the Standard Deviation.

a Proportion (%) of the chromosome sequence covered by methods.

**Table 4:** Number of methylation sites identified with CPSORL in chromosomes 21 and 22 of the human genome. (NCBI. 36).

*Chromosome number*	*21*	*22*
Chromosome length (bp)	46,944,323	49,691,432
Total length of CpG island (bp)	1,607,472	2,907,983
Number of methylation sites in entire genome	841,554	1,120,517
Number of methylation sites using CPSORL	111,172	185,324
Methylation density of CpG islands (%)	6.91	6.37

**Table 5:** Number of methylation sites identified with CPSORL in all chromosomes of the human genome. *(NCBI.36)*.

Chr.	Length	Total length of CpG island	Number of all methylation sites	Number of predicted methylation sites	Methylation Density (%)
1	247,249,719	9,819,708	5,006,940	523,354	5.33
2	242,951,149	7,822,751	5,023,026	431,279	5.51
3	199,501,827	5,561,406	3,965,121	310,656	5.58
4	191,273,063	5,331,470	3,577,143	275,413	4.95
5	180,857,866	5,780,736	3,563,532	318,252	5.51
6	170,899,992	5,858,975	3,465,347	318,445	5.44
7	158,821,424	6,784,935	3,450,658	392,566	5.79
8	146,274,826	4,841,004	3,015,121	267,302	5.52
9	140,273,252	5,384,493	2,574,014	282,008	5.23
10	135,374,737	5,245,458	3,013,632	292,186	5.57
11	134,452,384	5,228,058	2,872,470	282,971	5.41
12	132,349,534	5,512,364	2,957,221	195,079	3.54
13	114,142,980	3,049,962	1,946,147	180,554	5.92
14	106,368,585	3,536,154	1,935,241	191,968	5.43
15	100,338,915	3,676,992	1,858,038	186,212	5.06
16	88,827,254	5,414,278	2,222,494	320,771	5.92
17	78,774,742	6,551,708	2,306,666	252,464	3.85
18	76,117,153	2,528,076	1,605,879	180,108	7.12
19	63,811,651	7,604,015	1,939,151	461,782	6.07
20	62,435,964	3,106,557	1,551,541	180,108	5.80
21	46,944,323	1,607,472	841,554	111,172	6.91
22	49,691,432	2,907,983	1,120,517	185,324	6.37
X	154,913,754	4,831,155	2,279,012	190,792	3.95
Y	57,772,954	1,001,532	214,434	15,945	1.59
*Avg.*	128,350,812	4,957,802	2,596,037	264,446	5.33

**Table 6:** Comparison of different methods on the number of CpG islands identified in the entire human genomes.

*Methods*	*CpGcluster*	*CpGIS*	*CPSORL*	*CPSORL(Alu)*
Genome length	2.86E+09			
Number of predicted islands	198,702	37,729	208,536	54,483
*Coverage (%)*	1.90	1.44	4.1	2.1
Island length				
*Average*	273±246	1,090±717	572±469	1100±541
*GC content ±SD*	63.78±7.50	60.61±5.06	53.90±5.25	56.26±6.45
*O/E ratio ±SD*	0.855±0.265	0.717±0.082	0.649±0.087	0.665±0.10
TSSs	21,741 (10.9%)	15,106 (40.0%)	25,477 (12.2%)	22,057 (40.5%)
Promoter regions	29,156 (14.7%)	13,196 (35.0%)	54,356 (26.1%)	37,038 (67.8%)

## Discussion

### CpG island prediction performance in the contigs

We compared CPSORL with four other methods reported in the literature, namely CpGIS [3], CpGplot [4], CpGProD [5], CpGcluster [6] and PSO. Table 1 shows that the *SN* of the proposed method was highest on the NT_113952.1 (84.88%), NT_113955.2 (87.38%), NT_113958.2 (84.11%), NT_113953.1 (75.65%), NT_113954.1 (77.68%) and NT_028395.3 (77.02%) datasets (sensitivity bar graphs in Figure S3). The proposed method obtained better prediction results for CpG islands than the other methods tested. The accuracies (*ACC*) of CPSO and CPSORL are higher than the accuracies of the other methods. However, even though *ACC* of CPSORL is lower than *ACC* of CpGPlot in contig NT_113954.1, the *SN*, *PC* and *CC* of CPSORL are superior to CpGPlot. The reason for this is that CpGPlot does not obtain the *FP* in the search process, but rather yields many *FNs*. It therefore obtains high *SP* and *ACC* values and a lower *SN*. In addition, the performance of CPSO is better than that of CPSORL in the NT_113952.1 and NT_028395.3 contigs, the reason for this being that RL yields higher *FP* and lower *SP* values in the evaluation criteria. Hence, CPSO can obtain a high *CC*. As shown in Table 1, *SP* of this study is lower than the *SP* of CpGPlot in all contigs. CPSORL also showed the best *PC* and *CC* prediction performance on the chromosomes 21 and 22 contigs shown in Table 1, e.g., NT_113955.2 (87.89%), NT_113958.2 (79.31%), NT_113953.1 (73.10%) and NT_113954.1 (68.53%) have the highest *PC* and *CC* values. The *PC* can be viewed as a criterion to determine the method performance. The *CC* can be viewed as a combination of sensitivity and specificity [15]. In addition, we used the ROC curves for comparison in order to prove that CPSORL is superior to the other methods. An ROC curve is a plot of the false positive (FP) rate versus the true positive (TP) rate [16]. Figure S4 shows the ROC curves for all methods. Based on these plots it can be stated that the performance of CPSORL is better than the performance of the other methods it was compared to.

CpGIS, CpGplot and CpGProD all use the sliding window technique to predict CpG islands. These methods use the GC content, O/E ratio and length to predict CpG islands. These techniques are similar to brute force searches and thus yield high *SP* values. This causes the *SP* of some literature methods to be slightly higher than CPSORL; however, the difference generally lies below 1%. Sujuan *et. al.* [17] identified several disadvantages of the CpGIS, CpGplot and CpGProD methods: (1) CpG islands identified by these methods generally do not start and end with a CpG dinucleotide [18]. (2) The number and length of the CpG islands is obtained based on the window size and the step size. If the window is large, several short and loosely distributed CpG islands may merge into a larger one. (3) The run time for these methods is relatively long. Hackenberg *et. al* [19] mentioned that the window size has a profound effect on the quality of the CpG island prediction. CpGcluster predicts CpG islands based on the physical distance between CpG dinucleotides. Although CpGcluster can identify some short CpG clusters that are functional, its high false positive (*FP*) rate strongly limits its use in genome-wide or chromosome-wide searches for promoter-associated CpG clusters in vertebrate genomes [20].

In this study, the window sizes of CPSORL and the sliding window technique are different since the traditional methods use fixed lengths to search for CpG islands. However, the prediction results are affected by the fixed window sizes. Hence, we used a random length of 200 bp to 2000 bp based on the GGF criteria. This improved the prediction quality. As shown in Table 1, CPSORL was the most reliable and stable of the methods tested. It obtained the highest performance amongst all methods compared due to the combination of the improved evolutionary CPSO algorithm with RL.

### Improved CpG island search with CPSORL

We base our discussion in this section on the contig NT_113954.1 since this contig poses a challenge for most tools. When compared to the CpGIS tool, CPSORL provides equal or better prediction. CPSORL obtained higher prediction correlation coefficient (*CC*) than the other programs tested. The performance of CpGPlot, CpGcluster and CpGProd in identifying CpG islands is poorer than that of CpGIS, PSO and CPSO, as shown in Table S1. The CpGPlot, CpGcluster, and CpGProd software found a total of four, seven, and five CpG islands, respectively. However, CpGcluster found four CpG islands that did not meet the GGF criteria (two islands violated the O/E criterion, and two islands violated the length criterion). Three islands found by CpGProD were falsely identified because the O/E ratios did not meet the GGF criteria. Even though the total CpG islands length of CPSORL (6233 bp) is short than that of CpGIS (6647 bp), CpGIS found 19 CpG islands, of which six were falsely identified in the results. The 1^st^, 8^th^ and 10^th^ island had a GC content greater than 50%, and the 5^th^, 9^th^ and 11^th^ islands were longer than 200 bp, all conditions that violate the GGF criteria.

In Table S1, PSORL and CPSORL use RL to extend the total length from 4735 bp to 5835 bp and 5064 bp to 6233 bp, respectively. The island length increase is 23.2% and 23.1% for PSORL and CPSORL, respectively. In CPSORL for example RL extend the 2^nd^ and 6^th^ and CpG island from 202 bp and 1196 bp to 592 bp and 1356 bp, respectively. Furthermore, the complementary logic of CPSO increased the search capability of particles in the solution space, and thus the total length of the CpG islands increased by 1061 bp as compared to PSO. Since CpG islands are considered gene markers, the number of CpG islands should be close to the number of genes in the genome. At least half of the genes are overlapping with CpG islands in CPSORL. Associations of CpG islands with genes are shown in Table 2.

### CpG island prediction performance in chromosomes 21 and 22

Chromosomes 21 and 22 of the human genome are widely used in the literature, so we used the available data for these chromosomes to verify our results. Table 3 shows information pertaining to all the investigated methods for chromosomes 21 and 22, including the chromosome length, the number of islands predicted, the total length of the CpG islands, the island length (average, minimum, and maximum), the GC content, CpG island O/E ratio values and the coverage (%). The distribution of CpG islands (Figure S5) shows that most CpG islands lie in the region of 50–70% GC content and an O/E ratio of between 0.6 and 1.0, and thus the CpG islands conform to the GGF criteria. In addition, the CpG islands predicted in the entire human genome are shown in Figure S6.

Table 3 indicates that the number of CpG islands predicted by CpGIS is the highest for chromosomes 21 and 22. However, the total number of CpG islands does not represent a better prediction ability of this method since the average length of CpG islands predicted by CpGIS (346 bp and 413 bp for chromosome 21 and 22, respectively) is shorter than in our method (571 bp and 596 bp for chromosomes 21 and 22, respectively). CpGcluster predicted CpG islands with a minimum length as short as 8 bp. The total length of the CpG islands predicted in chromosomes 21 and 22 by CpGIS is shorter than in CPSORL. In addition, the high coverage means that the overlap region between the predicted CpG islands and the experimentally identified CpG islands is considerable and that the predicted CpG islands conform to the GGF criteria. When compared to the methods from the literature, the islands predicted by CPSORL covered a larger region (3.4% and 5.85%) in chromosomes 21 and 22, respectively.

RL is an intelligent system, which improves performance by receiving a feedback in the form of a scalar reward (or penalty) commensurate with the appropriateness of the response through the interactions of repeated tests and searches. If a known CpG island is separated into several predicted CpG island fragments, the prediction results are unreliable. In Table 1, the prediction performance of PSORL is generally lower than that of CpGIS. However, the performance of PSORL is higher than that of CpGIS (Table 3) in the chromosome 21. The reason may be that the RL system improves the prediction quality. The average of the GC content, O/E ratio and length values of CpG islands predicted by CPSORL and PSORL conform to the GGF criteria. Thus, the *SN* ratio of our method was higher than the *SN* ratio of the other methods.

DNA cytosine methylation plays an important role in biological processes, especially genome in mutation [21], embryonic development [22] and human diseases [23]. DNA methylation at CpG dinucleotides is a common feature in genomes. Generally, DNA methylation occurs in dinucleotide rich regions of the CpG islands. In general, around 80% of all CpG dinucleotides are methylated in mammalian genomes [24]. The lack of methylation is thus a very good indicator of the function of a CpG island [25]. We compared the methylation distribution on chromosomes 21 and 22 to the literature [26]. In this study, the methylation numbers in the CpG islands are 111,172 and 185,324 on chromosomes 21 and 22, respectively. The methylation densities in the CpG islands are shown in Table 4. The methylation densities of the CpG islands in this study are 6.9% and 6.37% on chromosomes 21 and 22, respectively. These results confirm the higher CpG island methylation densities predicted by the proposed method. Higher methylation densities exist in CpG islands of tumors [27] and cancer cells [28]. In this study, CPSORL was used to verify that the average methylation density was 5.33% for the CpG islands in entire human genome. The methylation number in the entire human genome and methylation calculated densities are shown in Table 5 and Figure S7. Results obtained by CPSORL imply that the methylation is present in CpG islands.

### CpG island prediction performance in all chromosomes

We compare the prediction performance of CpGIS, CpGcluster, CPSORL and CPSORL (*Alu*) in Table 6, which shows statistical values for the human genomes. The numbers for CPSORL are markedly larger than those for of CpGIS and CpGcluster. There are 208,536 islands predicted by CPSORL, 5.5 times the number of CpGIS (37,729) and 1.05 times the number of CpGcluster (198,702) [20]. The CpG island average length as determined by CPSORL is much longer than the average length given by CpGIS and CpGcluster. The average length determined by CPSORL is 572 bp where as CpGcluster yielded an average length of 273 bp. If the *Alu* sequences are considered in CPSORL, the CPSORL (*Alu*) length is 1100 bp, slightly longer than that of CpGIS 1090 bp. Both CPSORL and CPSORL (*Alu*) use RL to extend the length. Hence, on the O/E ratio and GC content average value are lower than for CpGIS and CpGcluster. This is due to the fact that CpGIS, CpGcluster and CPSORL use the length size difference. The minimum length of CpGcluster is 8 bp, the minimum length of CpGIS is 500 bp and for CPSORL it is 200∼2 Kbp. We compared the length distribution of CpGIS, CpGcluster, CPSORL and CPSORL (*Alu*) in the human genome. CPSORL determined most CpG islands to be in a range of 50∼500 bp long (66.34%). The length distribution of the CpG islands is shown in Figure S8.

In the study, we examined the promoter and transcription start site (TSS) overlap in CpG island region. A promoter region was defined as −1,500 bp to +500 bp bp around the TSS. In the human genome (Table 6), the CPSORL TSS number is higher (below 1.3%) than the TSS number of CpGcluster; the promoter region numbers are also higher (below 11.4%). If the *Alu* sequences are considered, the CPSORL (*Alu*) numbers are also higher than the TSS and promoter region numbers of CpGIS TSS and promoter regions. CPSORL (*Alu*) obtained 22,057 TSS and 37,038 promoter regions, whereas CpGIS obtained 15,106 TSS and 13,196 promoter regions. CpGcluster identified some short functional CpG clusters, but its high false positive rate strongly limits its use in genome-wide searches for promoter-associated CpG clusters in vertebrate genomes [20]. The CpG island values predicted by CPSORL(*Alu*) conform to the TJ criteria (length≧500 bp, GC content≧55% and O/E ratio≧0.65) [3].

## Materials and Methods

### Data Sets

From all available contigs we randomly selected contigs NT_113953.1, NT_113954.1, NT_113955.2, NT_113958.2 and NT_113952.1 in chromosome 21 and NT_028395.3 in chromosome 22. The contigs include the start and stop sites, transcription orientation and the evidence code as an example to illustrate the method. However, calculations were carried out for all the contigs in the human genome, which were extracted from NCBI (http://www.ncbi.nlm.nih.gov). The data of experimentally verified CpG islands was also extracted from NCBI.

### Particle Swarm Optimization (PSO)

PSO is a population-based stochastic optimization algorithm, which was developed by simulating the social behavior of organisms [11]. In PSO, each particle in the search space can be considered “an individual bird of a flock”; it moves its position based on its own knowledge and that of its neighbors. In other words, each particle uses its own memory and the knowledge of neighbors to find the best position (solution). In PSO, *pbest* is the best position of a particle amongst its own past iterations, as expressed by the highest fitness value. The best fitness value amongst all individual *pbest* values is called the global best (*gbest*). At each generation, the position and velocity of every particle is updated according to its own *pbest* and *gbest*. The update equations are shown below:
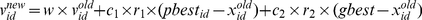
(6)

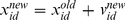
(7)where *r_1_* and *r_2_* are random numbers between (0, 1), and *C*_1_ and *C*_2_ are acceleration constants set to 2. Velocities *v^new^* and *v^old^* denote the velocities of the new and old particles, respectively. The positions *x^new^* and *x^old^* are the updated particle position and the current particle position, respectively.

An inertia weight *w* is used to control the balance between the global and local search. This weight is updated by the following equation:
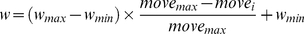
(8)where *w_max_* and *w_min_* are set to 0.9 and 0.4, respectively. *move_i_* and *move_max_* represent the current iteration number and the total number of iterations [11], respectively.

### Complementary Particle Swarm Optimization (CPSO)

**Figure 1:**
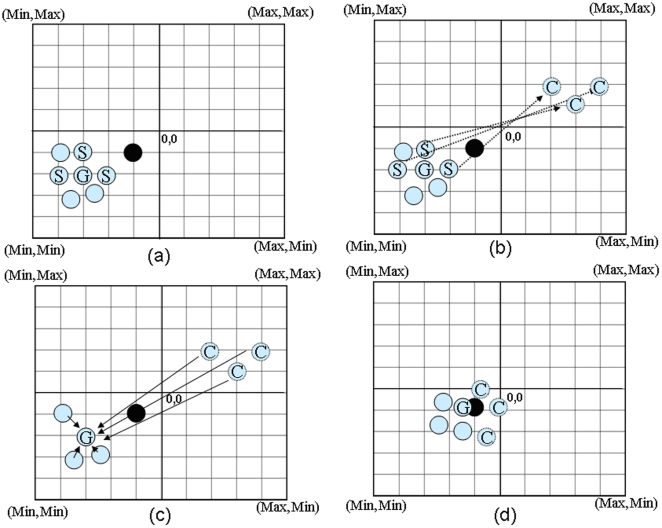
CPSO implementation diagram.

CPSO is an improved PSO algorithm, which uses a complementary concept to increase the performance of PSO. The aim is to improve the search ability of particles in the solution space, and to avoid particle entrapment in a locally optimal solution. When the distance of a particle to *gbest* is small, the particle proceeds with a local search. If *gbest* does not leave the local optimum after repeated iterations, a particle is considered trapped in a local optimum. Hence, we randomly select half of the optimal particle positions, and use the positions of the selected particles to generate complementary particles. The created complementary particles can escape the locally optimal solution and thereby increase the size of the search space. The following equation is used to create the complementary particles:
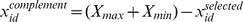
(9)Where *x^selected^* are the positions of randomly selected particles, and *x^complement^* are the positions of the complementary particles. *X_max_* and *X_min_* denote the maximum and minimum limit of the solution space, respectively. A diagram of the CPSO implementation is show in Figure 1. The entire process can be divided into four categories:

- When the *gbest* value is unchanged after five iterations, the program randomly regenerates half of the particles. In Fig. 1 (a), S and G represent the selected particles and *gbest*, respectively.
- The randomly selected particles S are replaced by the complementary particles, i.e., the C particles in the new search region, whereas not selected particles remain in the local search process.
- Since the location of the global optimum in a given area is unknown, the complementary particles are used to increase the search space based on the movement of *gbest*. However, if there are too many complementary particles, the search efficiency in the original search area is reduced. We therefore used 50% of the particles to achieve a balanced search. In addition, any complementary particles are also affected by the original *gbest* particle. Thus, CPSO tends to find a better position than the current *gbest* in the search process.
- Complementary particles have a fairly good chance of finding the globally optimal solution. The pseudo-codes for PSO and CPSO are shown in the Text S1.

### Reinforcement learning

Reinforcement learning (RL) constitutes an intelligence control system. It is characterized by effective, reactive, situational and adaptive properties and is robust under incomplete and uncertain knowledge of the domain. It is also perceptually feasible and based on mathematical foundations [10]. RL uses internal predictive models to improve the learning rate and tries out various output states to search for the best result. The result is evaluated repeatedly until a predefined criterion is reached. A RL system can be viewed as a machine whose unique target is to maximize the positive (correctness) and minimize the negative (errors). CpG islands that conform to the GGF criteria are predicted by PSO. However, the length of a predicted CpG island sequence is often shorter than the experimentally determined CpG island sequence. Hence, this study uses RL to extend the length of the predicted CpG islands. If the distance between adjacent CpG islands is smaller than 200 bp, the two CpG islands are combined. All predicted CpG islands are thus extended until the defined criterion is met. Examples for CpG islands with RL and CpG islands without RL are shown in Figure S1. The RL pseudo-code is also shown in the supplementary data section.

### Initialization

Since chromosome sequences are rather long, an input sequence is divided into window sections that continually predict CpG islands. The particle encoding is given by:
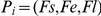

*i* is the particle number, *Fs* is the predicted start position of a CpG island, *Fe* is the predicted end position of a CpG island, and *Fl* is the predicted length of a CpG island.

### Fitness Evaluation

This study uses the GGF criteria (GC content≧50%, O/E ratio≧0.6, length ≧200 bp) to predict CpG islands in the human genome. The fitness functions of the length, the GC content and the O/E ratio are defined in Eqs. (10–12), respectively. In addition, Eq. (13) is used to calculate the fitness value of each particle.
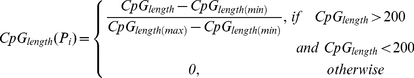
(10)

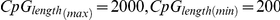


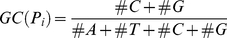
(11)

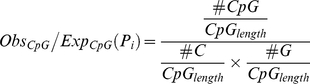
(12)

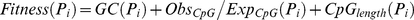
(13)Where the #A: number of A (Adenine), #T: number of T (Thymine), #C: number of C (Cytosine) and #G: number of G (Guanine) nucleotides in the CpG islands represented byparticle *P_i_*. #CpG: number of CpG islands. *CpG_length_*: length of CpG island.

A fitness function is used to evaluate the performance of CPSORL. A high fitness value means that CpG islands are predicted with high correlation coefficient and sensitivity. In general, the length of CpG islands is 200 bp–2000 bp. However, in order to reduce the fitness value of the CpG island length, a normalization function was used to adjust the fitness function. The length value is adjusted to a range of 0 to 1. A step-by-step description of the calculations performed by the algorithm is shown in Figure S9.

## Supporting Information

Figure S1 Comparison of CpG island prediction with and without reinforcement learning. The short bars indicate the CpG islands. (**A**) Without reinforcement learning, the known CpG islands are divided into two segments by the CPSO-RL prediction. (**B**) With reinforcement learning, a signal CpG islands is predicted by CPSO-RL that matches a real CpG island.
(DOC)

Figure S2 Illustration of calculating TP, TN, FP and FN. (TP, TN, FP and FN represent true positives, true negatives, false positives and false negatives, respectively.)
(DOC)

Figure S3 Bar graphs illustrating the different sensitivities for each method on chromosomes 21 and chromosome 22 contigs.
(DOC)

Figure S4 ROC curves plotted for all methods to evaluate the data sets.
(DOC)

Figure S5 Distribution of CpG islands in the entire human genome. The blue dots indicate the CpG islands, and the x and y axes are the GC% and the CpGs *o/e* ratio, respectively. Most CpG islands lie in the region of 50–70% GC, and an *o/e* ratio of between 0.6 and 1.0.
(DOC)

Figure S6 Analysis of predicted CpG islands by CPSORL in the entire human genome.
(DOC)

Figure S7 Illustration of calculating methylation densities.
(DOC)

Figure S8 Length distribution of CPSORL and other methods in the human genome.
(DOC)

Figure S9 A description of the step-by-step procedures for the algorithm.
(DOC)

Text S1 The pseudo-codes for PSO and CPSO.
(DOC)

Table S1 Comparison of different CpG island prediction tools for contig NT_113954.1.
(DOC)
