# Supplementary material for: Particle Swarm Optimization with Reinforcement Learning for the Prediction of CpG Islands in the Human Genome
Source: PLoS One. 2011 Jun 28;6(6):e21036. doi: 10.1371/journal.pone.0021036 (PMC3125183; doi:10.1371/journal.pone.0021036)
Supplement: Figure S1 — Comparison of CpG island prediction with and without reinforcement learning. The short bars indicate the CpG islands. (A) Without reinforcement learning, the known CpG islands are divided into two segments by the CPSO-RL prediction. (B) With reinforcement learning, a signal CpG islands is predicted by CPSO-RL that matches a real CpG island. (DOC) [file pone.0021036.s001.doc]

**Figure S1.**


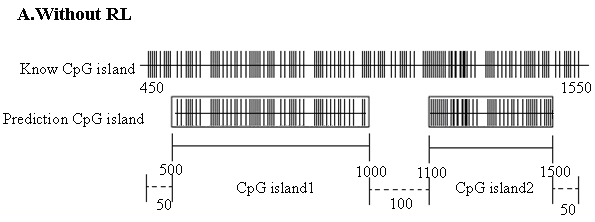


Fig. 1A shows that for a known CpG island located at 450 bp~1550 bp, 2 CpG islands are predicted (CpG island1: 500~1000 bp and CpG island2: 1100~1500bp) when RL is not used. This fact reduces the *SN* value.

**
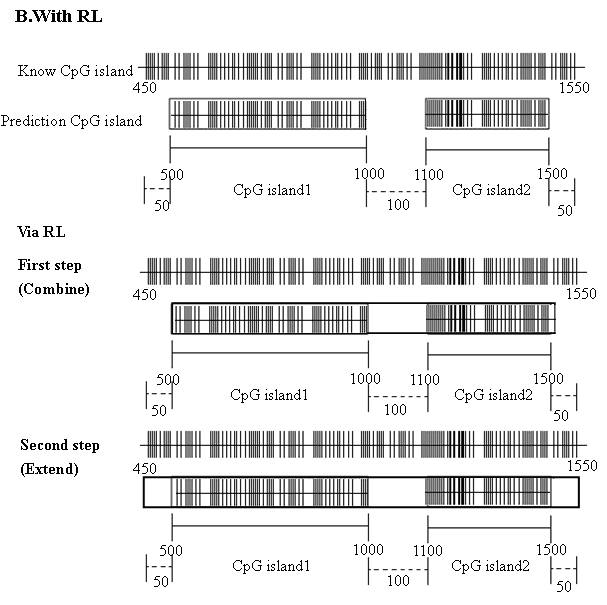
**

Fig. 1B. First step: RL is used to combine the CpG islands to avoid separation of a single known CpG island into separate islands. Second step: the length of the known CpG island is extended and thus *FN* is reduced and *SN* improved.
