# Supplementary material for: Particle Swarm Optimization with Reinforcement Learning for the Prediction of CpG Islands in the Human Genome
Source: PLoS One. 2011 Jun 28;6(6):e21036. doi: 10.1371/journal.pone.0021036 (PMC3125183; doi:10.1371/journal.pone.0021036)
Supplement: Figure S2 — Illustration of calculating TP, TN, FP and FN. (TP, TN, FP and FN represent true positives, true negatives, false positives and false negatives, respectively.) (DOC) [file pone.0021036.s002.doc]

**Figure S2.**


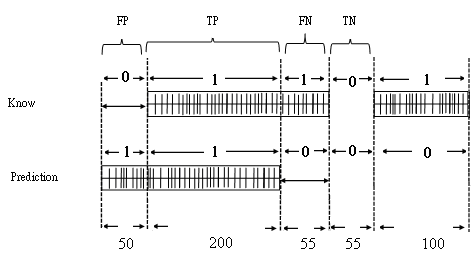


Example 1:

Example island length=50+200+55+55+100=460

Positive=1,Negative=0

| Know  Prediction | Negative  (0) | Positive  (1) |
| --- | --- | --- |
| Negative (0) | TN | FN |
| Positive (1) | FP | TP |

TN = (know island=0 and prediction=0) = 55

FP = (know island=0 and prediction=1) = 50

FN = (know island=1 and prediction=0) = 55+100

TP = (know island=1 and prediction=1) = 200

Length= TN +FP+FN+TP=55+50+55+100+200=460

Length= example island length (Check for CpG island position overlapp)

$$SN=\frac{TP}{TP+FN}=\frac{200}{200+(55+100)}=0.563$$

$$SP=\frac{TN}{TN+FP}=\frac{55}{55+50}=0.523$$

$$ACC=\frac{TP+TN}{TP+FP+TN+FN}=\frac{200+55}{200+50+55+(55+100)}=0.554$$

$$PC=\frac{TP}{TP+FN+FP}=\frac{200}{200+(55+100)+50}=0.655$$

$$\begin{matrix} CC=\frac{TP*TN-FP*FN}{\sqrt{(TP+FN)*(TP+FP)*(TN+FN)*(TN+FP)}} \\ \text{ }=\frac{200*55-50*(55+100)}{\sqrt{(200+55+100)*(200+50)}*(55+55+100)*(55+50)} \\ \text{ }=0.073 \end{matrix}$$

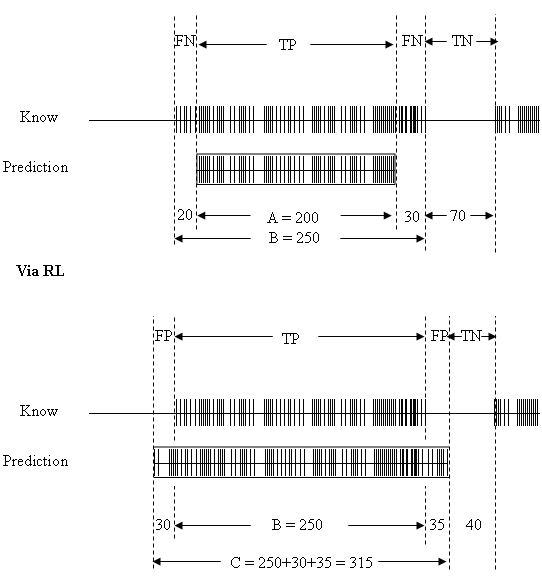


Example (Original without RL):

| $SN=\frac{TP}{TP+FN}=\frac{200}{200+(20+30)}=\frac{200}{250}=0.8$ |
| --- |
| $SP=\frac{TN}{TN+FP}=\frac{70}{70+0}=1$ |
| $ACC=\frac{TP+TN}{TP+FP+TN+FN}=\frac{200+70}{200+0+70+(20+30)}=\frac{270}{320}=0.843$ |
| $PC=\frac{TP}{TP+FN+FP}=\frac{200}{200+(20+30)+0}=\frac{200}{250}=0.8$ |
| $\begin{matrix} CC=\frac{TP*TN-FP*FN}{\sqrt{(TP+FN)*(TP+FP)*(TN+FN)*(TN+FP)}} \\ \text{ }=\frac{200*70-0*(20+30)}{\sqrt{(200+(20+30))*(200+0)*(70+20+30)*(70+0)}}=\frac{14000}{\sqrt{420000000}}=0.683 \end{matrix}$ |

Example (RL):

| $SN=\frac{TP}{TP+FN}=\frac{250}{250+0}=\frac{250}{250}=1$ |
| --- |
| $SP=\frac{TN}{TN+FP}=\frac{40}{40+(30+35)}=\frac{40}{105}=0.38$ |
| $ACC=\frac{TP+TN}{TP+FP+TN+FN}=\frac{250+40}{250+(30+35)+40+0}=\frac{290}{355}=0.816$ |
| $PC=\frac{TP}{TP+FN+FP}=\frac{250}{250+0+(30+35)}=\frac{250}{315}=0.793$ |
| $\begin{matrix} CC=\frac{TP*TN-FP*FN}{\sqrt{(TP+FN)*(TP+FP)*(TN+FN)*(TN+FP)}} \\ \text{ }=\frac{250*40-(30+35)*0}{\sqrt{(250+0)*(250+(30+35)*(40+0)*(40+(30+35))}}=\frac{10000}{\sqrt{330750000}}=0.554 \end{matrix}$ |

Table 1. Comparison of original prediction and via RL for CpG island

|  | Original | Via RL |
| --- | --- | --- |
| SN | 0.8 | 1 |
| SP | 1 | 0.38 |
| ACC | 0.843 | 0.816 |
| PC | 0.8 | 0.793 |
| CC | 0.683 | 0.554 |

The advantages of RL can improve the overall performance. However there is also one drawback: when RL extension is outdone, unnecessary *FP* are predicted. Although the extended CpG island are still consistent with GGF criteria, the *SP*, *ACC*, *PC*, and *CC* are all reduced.
