# Supplementary figures and images for: Particle Swarm Optimization with Reinforcement Learning for the Prediction of CpG Islands in the Human Genome
Source: PLoS One. 2011 Jun 28;6(6):e21036. doi: 10.1371/journal.pone.0021036 (PMC3125183; doi:10.1371/journal.pone.0021036)

**Figure S3.**

**
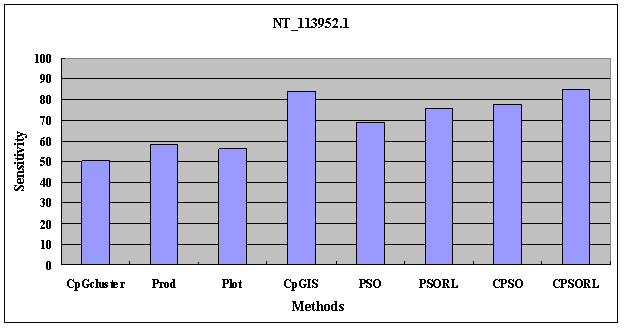
**

**
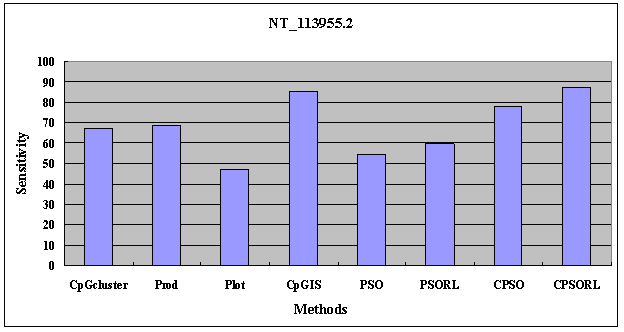
**


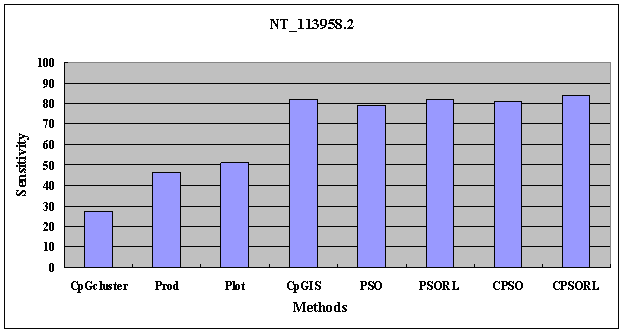


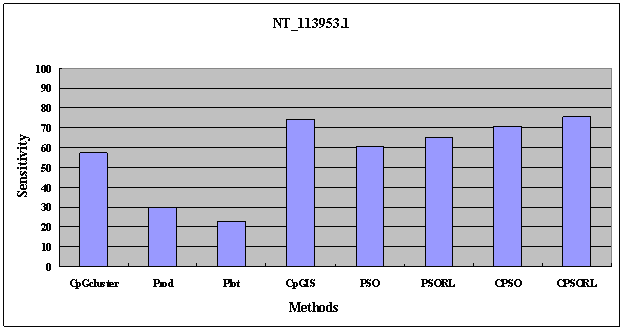


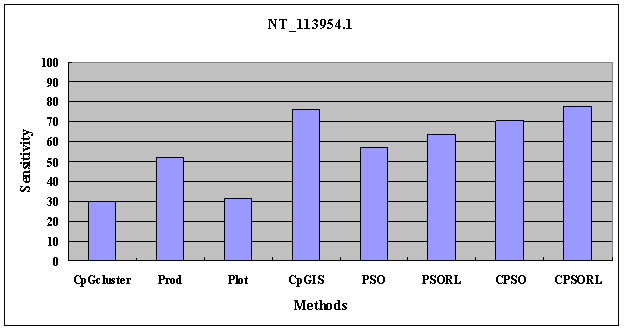


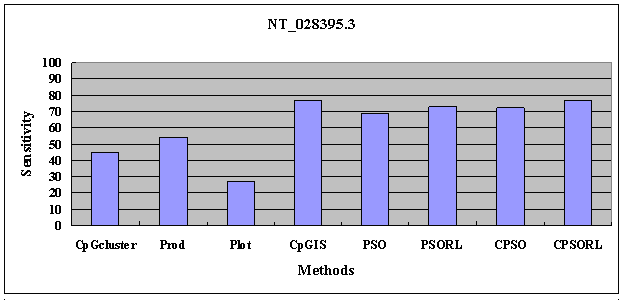

Supplement: Figure S3 — Bar graphs illustrating the different sensitivities for each method on chromosomes 21 and chromosome 22 contigs. (DOC) [file pone.0021036.s003.doc]

**Figure S4.**

**
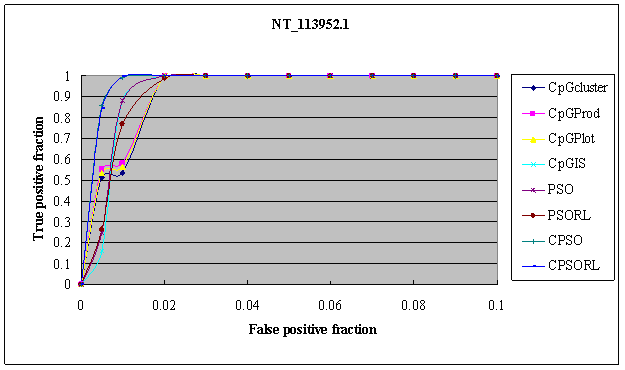
**

**
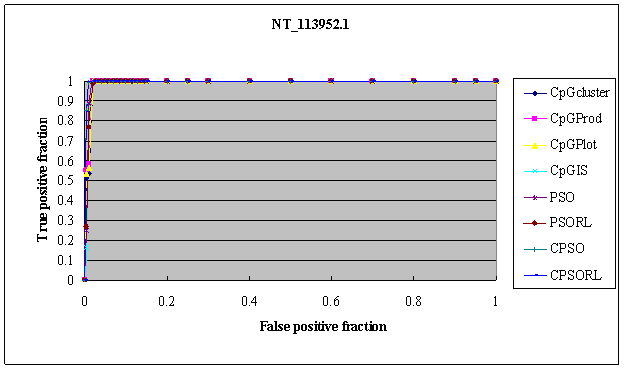
**

**
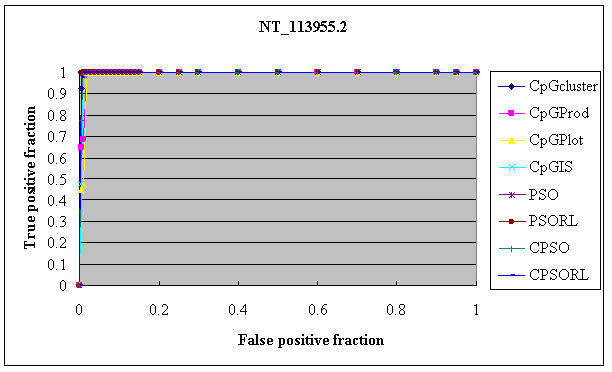
**

**
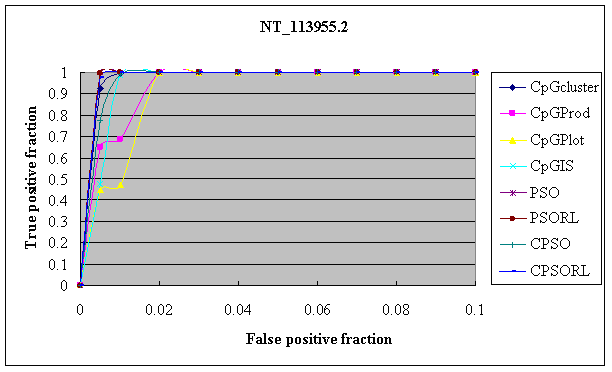
**

**
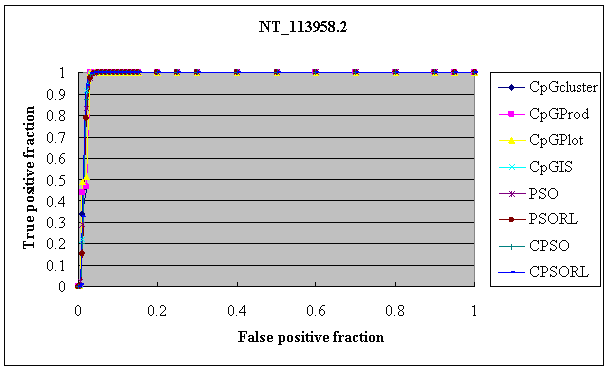
**


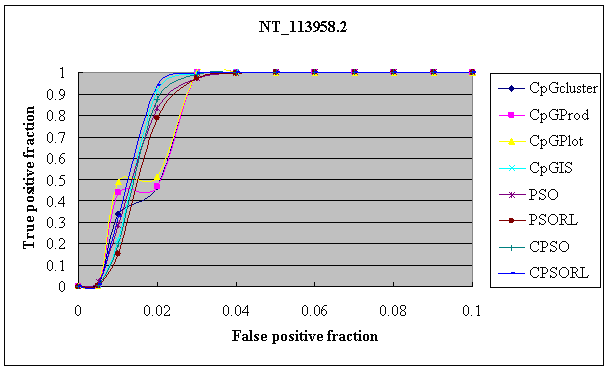


**
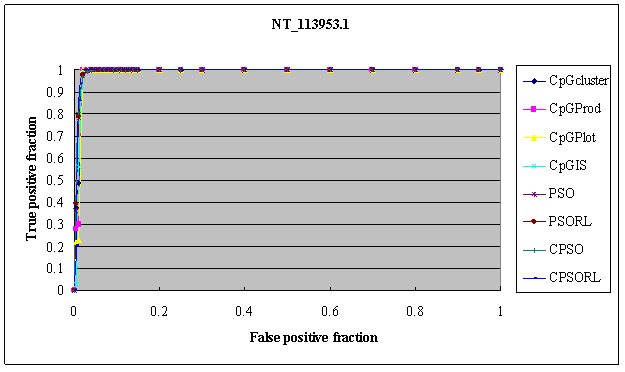
**

**
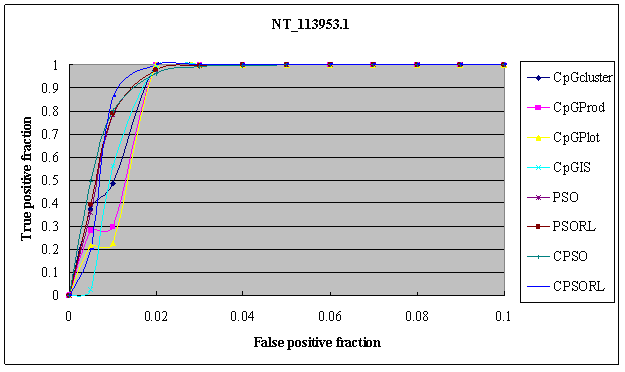
**

**
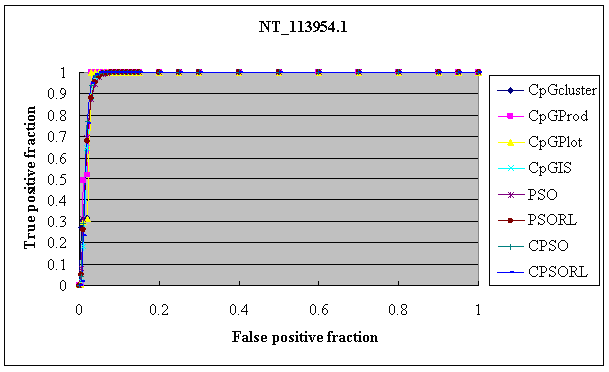
**

**
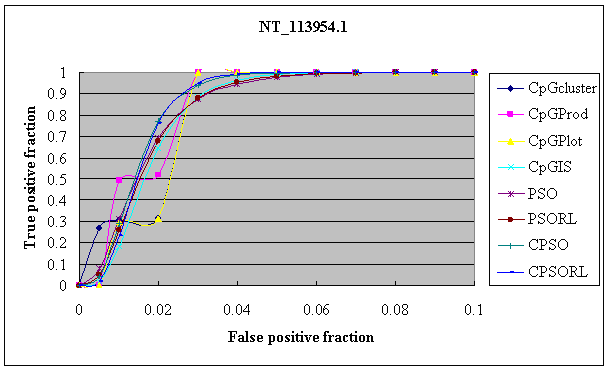
**

**
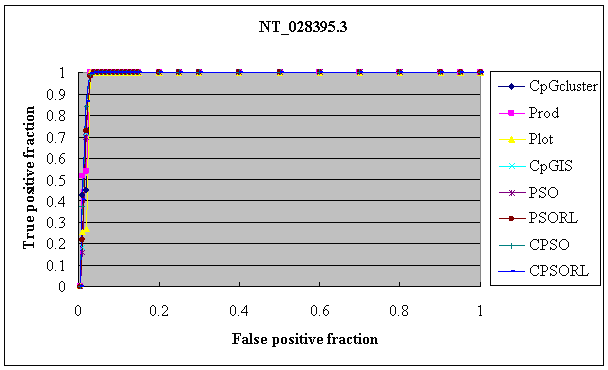
**

**
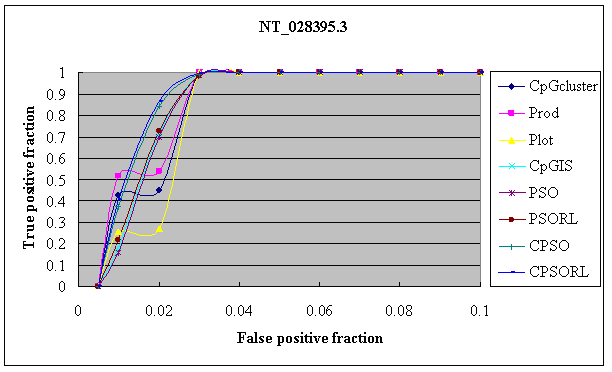
**

Supplement: Figure S4 — ROC curves plotted for all methods to evaluate the data sets. (DOC) [file pone.0021036.s004.doc]

**Figure S5.**

**
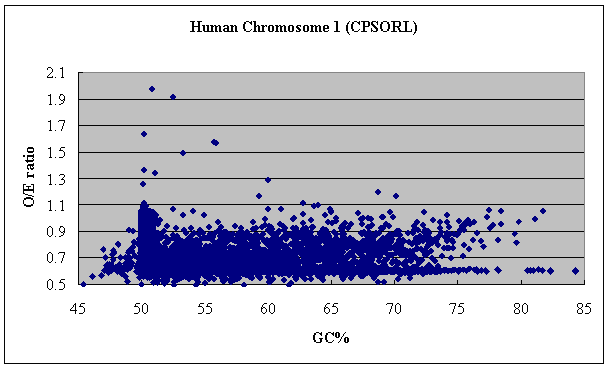
**


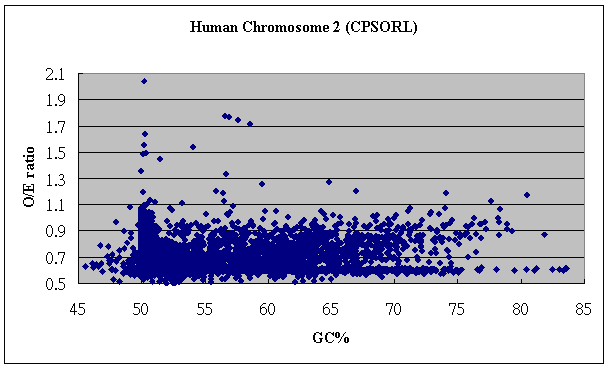


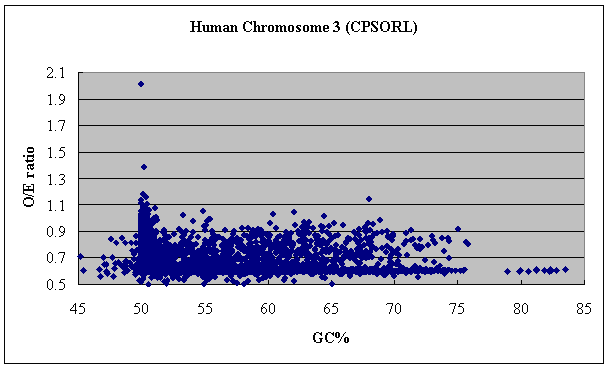


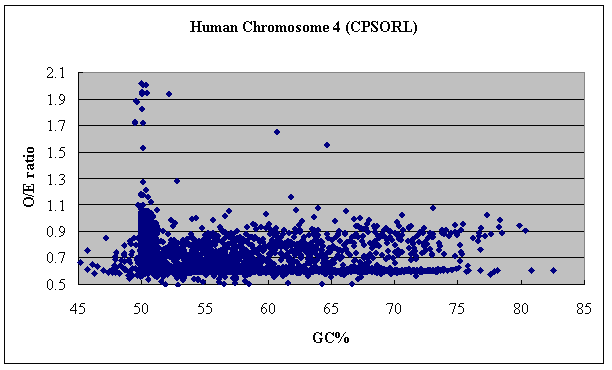


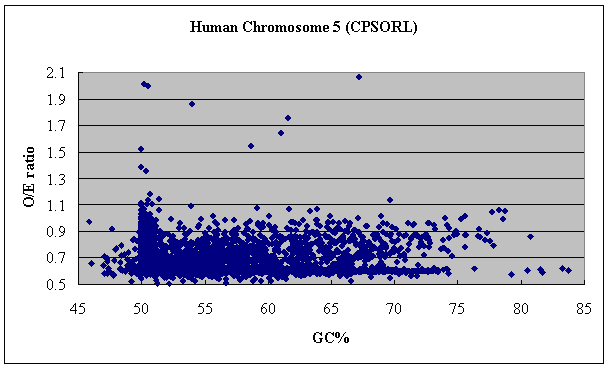


**
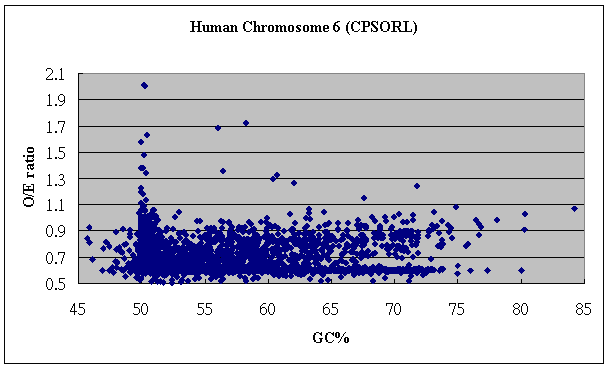
**

**
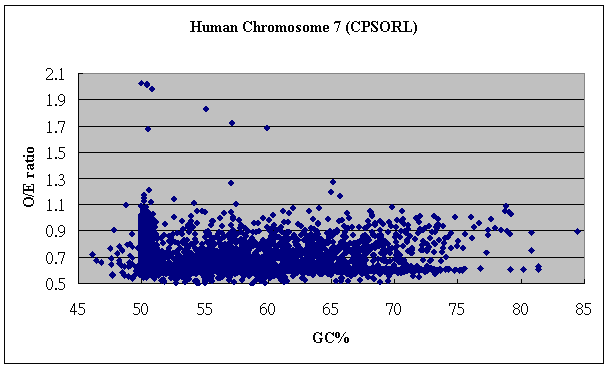
**

**
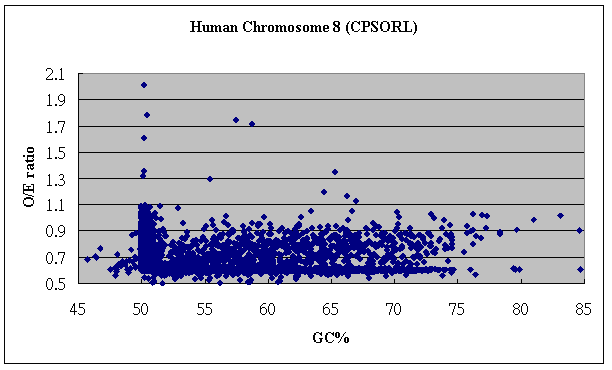
**

**
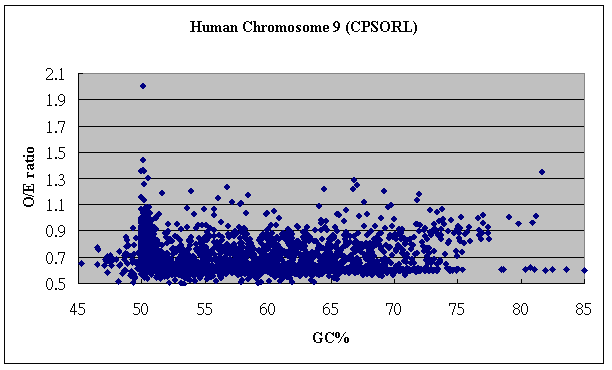
**

**
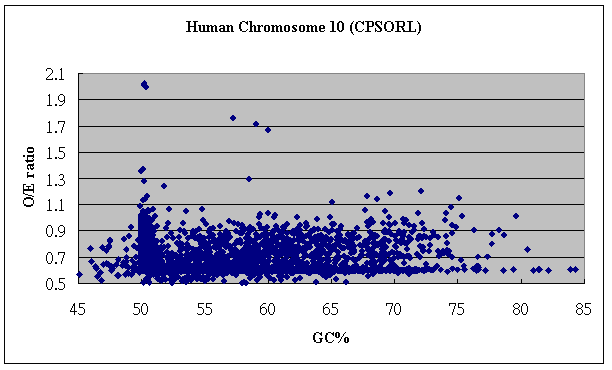
**

**
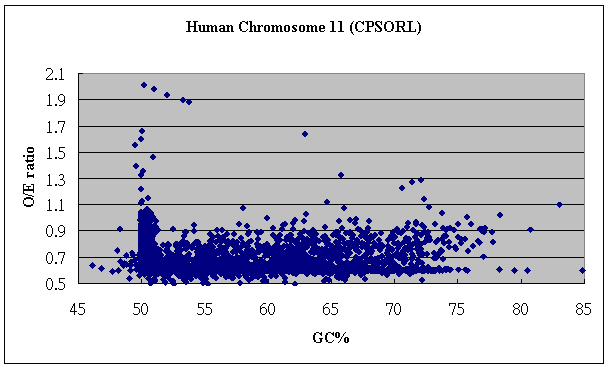
**


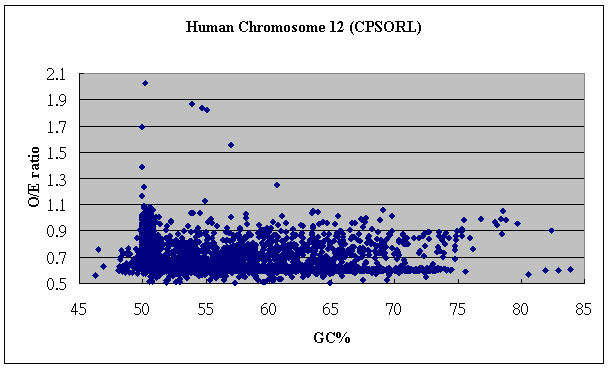


**
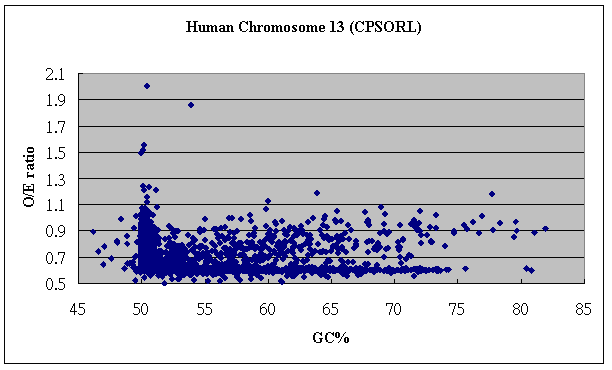
**

**
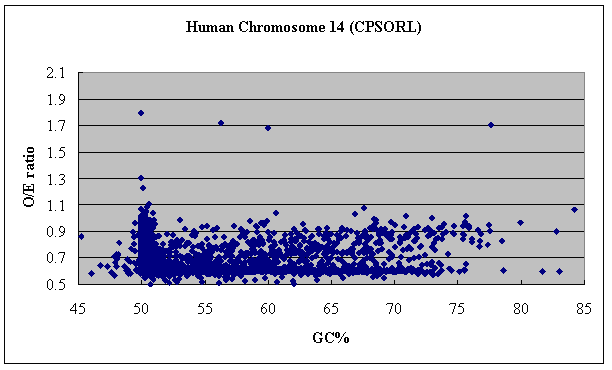
**

**
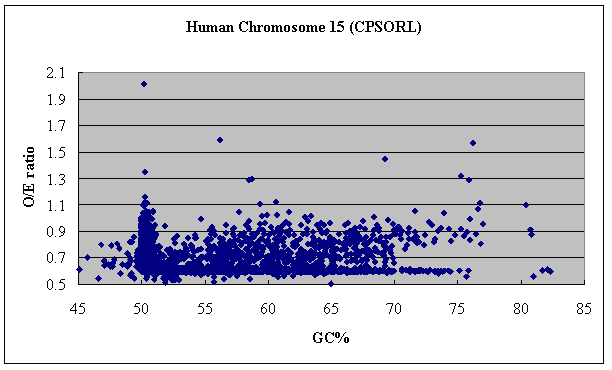
**

**
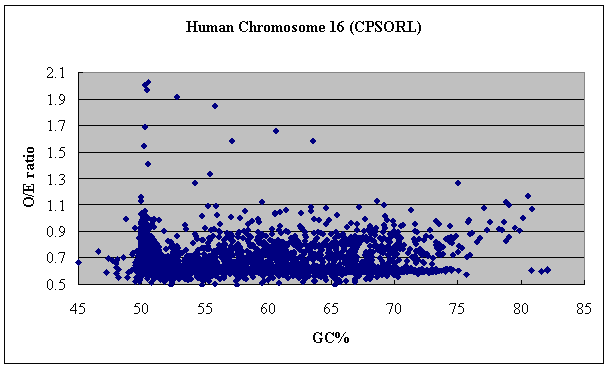
**

**
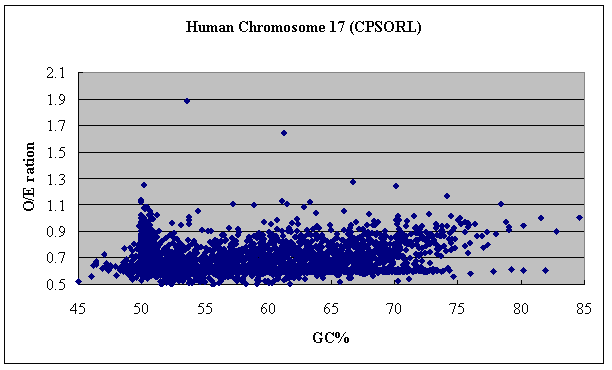
**

**
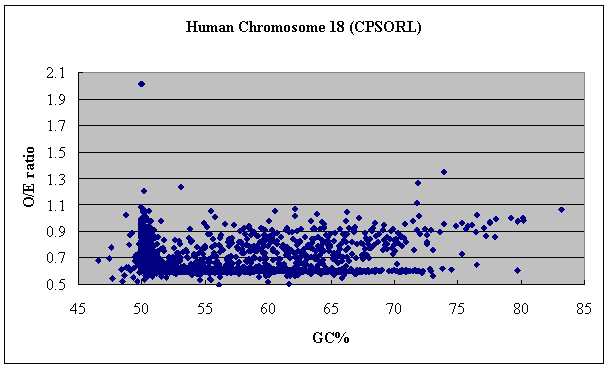
**

**
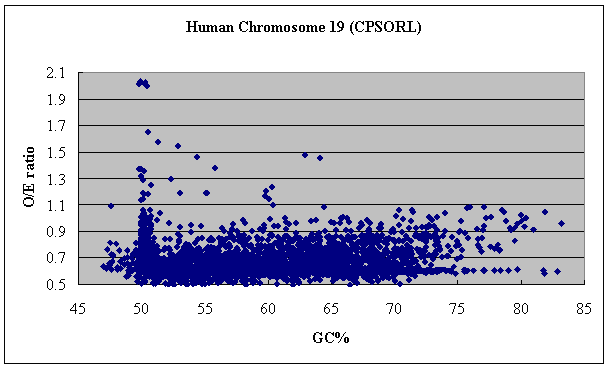
**

**
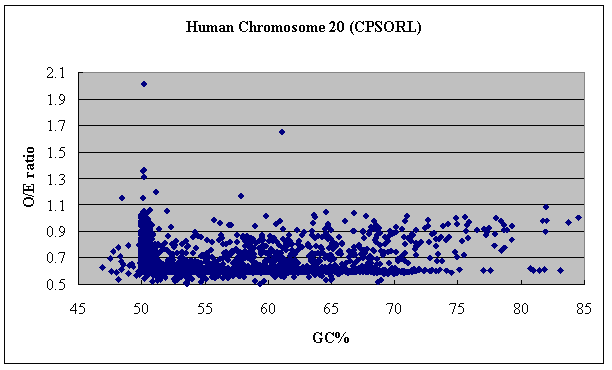
**

**
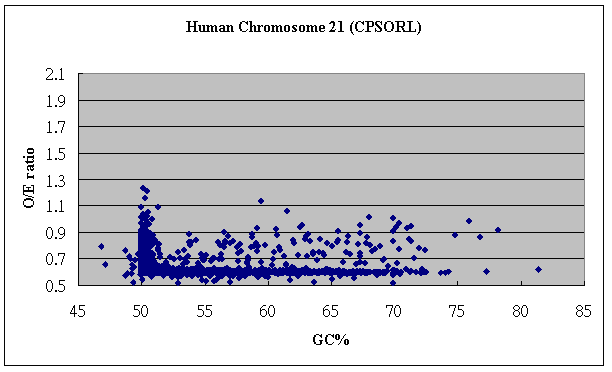
**

**
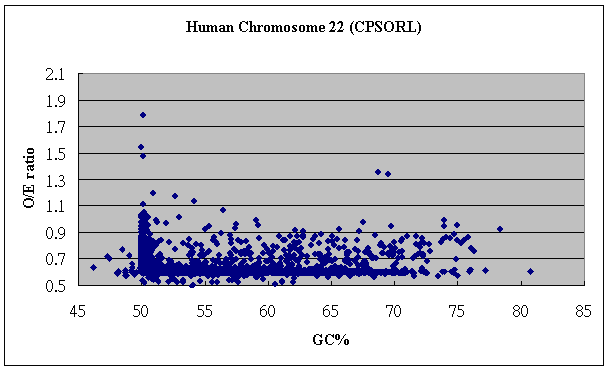
**

**
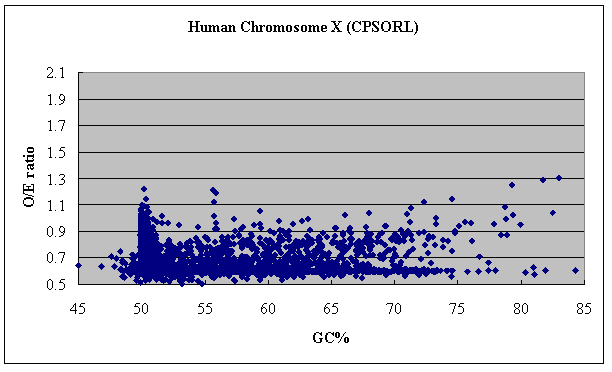
**


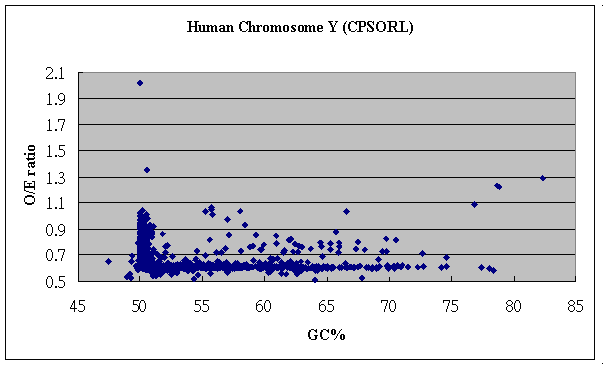

Supplement: Figure S5 — Distribution of CpG islands in the entire human genome. The blue dots indicate the CpG islands, and the x and y axes are the GC% and the CpGs o/e ratio, respectively. Most CpG islands lie in the region of 50–70% GC, and an o/e ratio of between 0.6 and 1.0. (DOC) [file pone.0021036.s005.doc]

**Figure S8.**


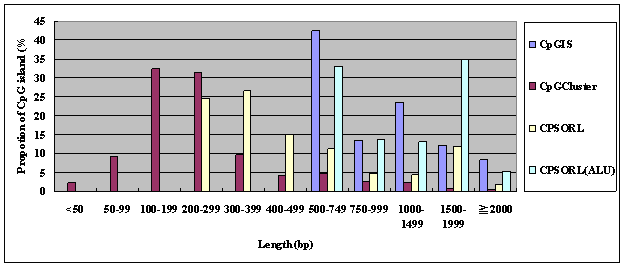


Length distribution of CPSORL and other methods in the human genome.

Supplement: Figure S8 — Length distribution of CPSORL and other methods in the human genome. (DOC) [file pone.0021036.s008.doc]
