# Supplementary material for: Particle Swarm Optimization with Reinforcement Learning for the Prediction of CpG Islands in the Human Genome
Source: PLoS One. 2011 Jun 28;6(6):e21036. doi: 10.1371/journal.pone.0021036 (PMC3125183; doi:10.1371/journal.pone.0021036)
Supplement: Figure S6 — Analysis of predicted CpG islands by CPSORL in the entire human genome. (DOC) [file pone.0021036.s006.doc]

**Figure S6.**

Analysis of CpG islands predicted by CPSORL in the entire human genome.

| ***Chr.*** | ***Number of predicted islands*** | ***Mean length*** | ***GC%±SD*** | ***CpGs o/e ratio±SD*** |
| --- | --- | --- | --- | --- |
| 1 | 16,932 | 579.9 | 54 ± 5 | 0.64±0.08 |
| 2 | 14,179 | 551.7 | 53 ± 4 | 0.65±0.09 |
| 3 | 10,435 | 532.9 | 53 ± 4 | 0.65±0.08 |
| 4 | 9,749 | 546.8 | 53 ± 5 | 0.66±0.10 |
| 5 | 10,118 | 571.3 | 53 ± 5 | 0.66±0.09 |
| 6 | 10,321 | 567.7 | 53 ± 5 | 0.65±0.09 |
| 7 | 12,016 | 564.6 | 54 ± 5 | 0.65±0.09 |
| 8 | 8,555 | 565.9 | 54 ± 5 | 0.65±0.09 |
| 9 | 9,692 | 555.5 | 54 ± 5 | 0.65±0.09 |
| 10 | 9,238 | 567.8 | 54 ± 5 | 0.65±0.09 |
| 11 | 9,019 | 579.6 | 55 ± 6 | 0.65±0.09 |
| 12 | 10,147 | 543.2 | 54 ± 5 | 0.65±0.08 |
| 13 | 5,525 | 552.0 | 53 ± 5 | 0.66±0.10 |
| 14 | 6,179 | 572.2 | 54 ± 5 | 0.65±0.09 |
| 15 | 6,238 | 589.5 | 54 ± 5 | 0.65±0.09 |
| 16 | 9,198 | 588.6 | 55 ± 6 | 0.64±0.08 |
| 17 | 11,006 | 595.3 | 55 ± 6 | 0.64±0.07 |
| 18 | 4,562 | 554.2 | 53 ± 5 | 0.66±0.09 |
| 19 | 12,111 | 627.9 | 56 ± 6 | 0.63±0.08 |
| 20 | 5,396 | 575.7 | 55 ± 6 | 0.64±0.08 |
| 21 | 2,813 | 571.4 | 54 ± 5 | 0.65±0.09 |
| 22 | 4,882 | 595.7 | 54 ± 5 | 0.63±0.07 |
| X | 8,616 | 560.7 | 53 ± 5 | 0.65±0.08 |
| Y | 1,609 | 622.5 | 54 ± 5 | 0.65±0.10 |
| *Avg.* | 8,689 | 572 | 54 ± 5 | 0.65±0.09 |

**Legends:** *Chr.* indicates the chromosome, SD indicates Standard Deviation, CpGs *o/e* ration means observed/expected.

Analysis of CpG islands predicted by CPSORL (ALU) in the entire human genome.

| ***Chr.*** | ***Number of predicted islands*** | ***Mean length*** | ***GC%±SD*** | ***CpGs o/e ratio±SD*** |
| --- | --- | --- | --- | --- |
| 1 | 4,902 | 1119.6 | 57 ± 6 | 0.67±0.09 |
| 2 | 3,670 | 1090.6 | 56 ± 6 | 0.67±0.10 |
| 3 | 2,386 | 1070.1 | 55 ± 6 | 0.67±0.09 |
| 4 | 2,331 | 1075.9 | 55 ± 6 | 0.68±0.10 |
| 5 | 2,596 | 1122.4 | 56 ± 6 | 0.68±0.10 |
| 6 | 2,714 | 1102.7 | 55 ± 6 | 0.68±0.10 |
| 7 | 3,278 | 1088.3 | 56 ± 6 | 0.66±0.10 |
| 8 | 2,143 | 1105.7 | 57 ± 7 | 0.67±0.10 |
| 9 | 2,530 | 1066.4 | 57 ± 6 | 0.67±0.10 |
| 10 | 2,343 | 1122.5 | 55 ± 6 | 0.67±0.10 |
| 11 | 2,438 | 1115.6 | 56 ± 6 | 0.66±0.11 |
| 12 | 2,651 | 1029.9 | 56 ± 6 | 0.66±0.10 |
| 13 | 1,221 | 1062.5 | 55 ± 6 | 0.68±0.11 |
| 14 | 1,458 | 1122.3 | 57 ± 7 | 0.67±0.10 |
| 15 | 1,494 | 1167.6 | 56 ± 6 | 0.68±0.11 |
| 16 | 2,647 | 1114.6 | 58 ± 7 | 0.66±0.10 |
| 17 | 3,262 | 1124.7 | 57 ± 7 | 0.66±0.09 |
| 18 | 925 | 1083.8 | 57 ± 7 | 0.68±0.11 |
| 19 | 4,293 | 1099.5 | 56 ± 7 | 0.65±0.09 |
| 20 | 1,256 | 1126.9 | 58 ± 6 | 0.65±0.10 |
| 21 | 434 | 1089.6 | 58 ± 7 | 0.64±0.09 |
| 22 | 1,153 | 1144.1 | 56 ± 7 | 0.64±0.08 |
| X | 2,144 | 1092.1 | 56 ± 6 | 0.65±0.09 |
| Y | 214 | 1055.8 | 54 ± 5 | 0.63d±0.08 |
| *Avg.* | 2,270 | 1099.7 | 56 ± 6 | 0.67±0.10 |

**Legends:** *Chr.* indicates the chromosome, SD indicates Standard Deviation, CpGs *o/e* ration means observed/expected.
