# Supplementary material for: Particle Swarm Optimization with Reinforcement Learning for the Prediction of CpG Islands in the Human Genome
Source: PLoS One. 2011 Jun 28;6(6):e21036. doi: 10.1371/journal.pone.0021036 (PMC3125183; doi:10.1371/journal.pone.0021036)
Supplement: Figure S7 — Illustration of calculating methylation densities. (DOC) [file pone.0021036.s007.doc]

**Figure S7.**

The methylation density represents the percentage of sites that were methylated in the predicted CpG islands.

The calculation can be elucidated by the following example:


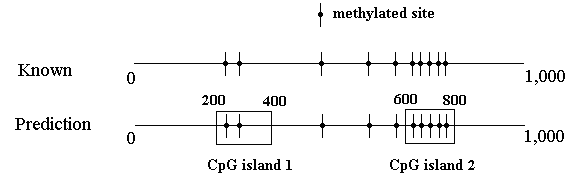


Given is a 1,000 bp long sequence that is known to contain 10 methylation sites. Suppose that the predicted CpG island1 (*length=200bp*) has 2 known methylation sites and the predicted CpG island2 (*length=200bp*) has 5 known methylation sites. We can then calculate the methylation density as:

$\frac{\text{Number of predicted methylation}}{\text{Total length of CpG island}}*100\%=\frac{2+5}{200+200}*100\%=1.75\%$

The methylation density is thus 1.75%.
