# Supplementary material for: Particle Swarm Optimization with Reinforcement Learning for the Prediction of CpG Islands in the Human Genome
Source: PLoS One. 2011 Jun 28;6(6):e21036. doi: 10.1371/journal.pone.0021036 (PMC3125183; doi:10.1371/journal.pone.0021036)
Supplement: Figure S9 — A description of the step-by-step procedures for the algorithm. (DOC) [file pone.0021036.s009.doc]

**Figure S9.**

**A step-by-step description of the proposed algorithm is given below.**


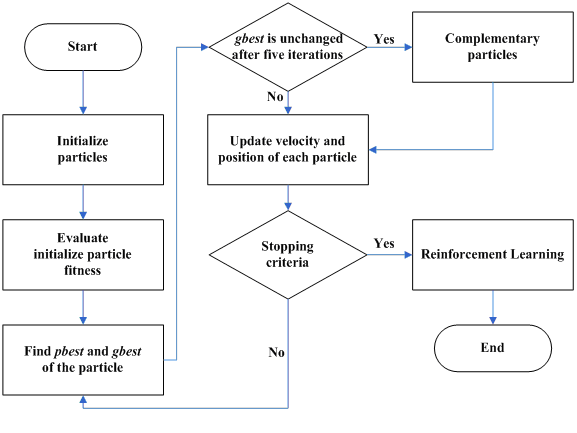


Example:

To predict CpG islands, each particle is encoded as *P_i_= (Fs, Fe)*, where *Fs* and *Fe* represent the start and end positions of a CpG island, respectively. In the example below, the population size is 3, and *C*_1_ and *C*_2_ are set to 2. The sequence length is 10,000 bp, i.e., we limit *Fe* to 10,000.


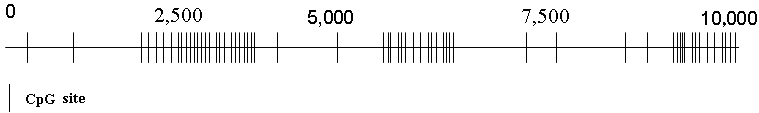


Step 1: particle initialization

*P_1_ = (0, 2000)*

*P_2_ = (2500, 4000)*

*P_3_ = (5100, 6200)*

Step 2: Evaluate fitness of *P­_i_* by using Eq. (1-4)

| *CpG_length(max)_=2000, CpG_length(min)_=200* | (1) |
| --- | --- |
| $GC(P_{i})=\frac{\#C+\#G}{\#A+\#T+\#C+\#G}$ | (2) |
| $\text{Obs}_{\text{CpG}}\text{/Exp}_{\text{CpG}}\text{ }(P_{i})=\frac{\frac{\#CpG}{CpG_{length}}}{\frac{\#C}{CpG_{length}}\times\frac{\#G}{CpG_{length}}}$ | (3) |
| $Fitness(P_{i})=GC(P_{i})+Obs_{CpG}/Exp_{CpG}(P_{i})+CpG_{length} (P_{i})$ | (4) |

#A: number of A (Adenine), #T: number of T (Thymine), #C: number of C (Cytosine) and #G: number of G (Guanine) nucleotides in the CpG islands represented by particle *P­_i_*. #CpG: number of CpG islands. *CpG_length_*: length of CpG island.

Step 3: Evaluate fitness of each particle *P_i_*


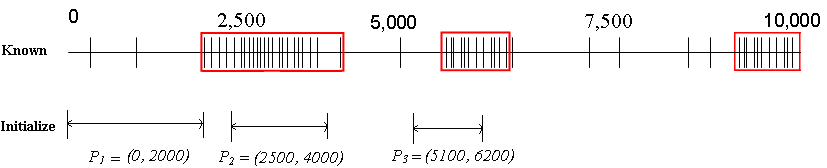


*P_1_ = (0, 2000),* the length of CpG island (*CpG_length_*) is 2,000 (2000-0) bp. If the number of C (*#C*) is 250, the number of G (*#G*) is 325 and the number of CpG (*#CpG*) is 8. The fitness (*P_1_*) is thus calculated as:

$$Fitness(P_{1})=\frac{250+325}{2000}+\frac{\frac{8}{2000}}{\frac{250}{2000}*\frac{325}{2000}}+\frac{(2000-0)-200}{2000-200}=0.287+0.197+1=1.48$$

*P_2_ = (2500, 4000),* the length of CpG island (*CpG_length_*) is 1,500 (4000-2500) bp. If the number of C (*#C*) is 750, the number of G (*#G*) is 700 and the number of CpG (*#CpG*) is 280. The fitness (*P_2_*) is thus calculated as:

$$Fitness(P_{2})=\frac{750+700}{1500}+\frac{\frac{280}{1500}}{\frac{750}{1500}*\frac{700}{1500}}+\frac{(4000-2500)-200}{2000-200}=0.96+0.8+0.72=2.48$$

*P_3_ = (5100, 6200),* the length of CpG island (*CpG_length_*) is 1,100 (6200-5100) bp. If the number of C (*#C*) is 500, the number of G (*#G*) is 450 and the number of CpG (*#CpG*) is 200. The fitness (*P_3_*) is thus calculated as:

$$Fitness(P_{3})=\frac{500+450}{1100}+\frac{\frac{200}{1100}}{\frac{500}{1100}*\frac{450}{1100}}+\frac{(6200-5100)-200}{2000-200}=0.86+0.98+0.5=2.34$$

The fitness of *pbest_1_* is 1.48, the fitness of *pbest_2_* is 2.48 and the fitness of *pbest_3_* is 2.34. Since the fitness value of *P_2_* is the maximum value, *pbest* is now the *gbest*: *gbest =pbest_2._*

*
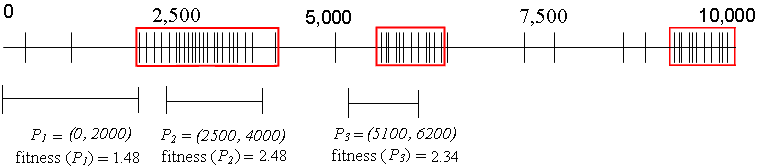
*

Step 4: If *gbest* has not improved for five iterations then half of the population is randomly selected and replaced by complementary particles to increase the search space.

Suppose that *P_1_*= (0, 2000) is selected; we use Eq. (5) to generate the complementary particle.

$x_{id}^{complement}=(X_{\max}+X_{\min})-x_{id}^{selected}$ (5)

where$x_{id}^{selected}$is the position of the randomly selected particle, and $x_{id}^{complement}$is the position of the respective complementary particle.$X_{\max}$and$X_{\min}$denote the maximum (10000,10000) and minimum (0,0) limit of the solution space, respectively.

$$\text{P}_{1}^{complement}=[(10000,10000)+(0,0)]-(0,2000)=(10000,\text{ }8000)$$

where 10000 represents the start site, and 8000 represents the end site. Since the start site must be in front of the end site, we change the search direction such that *P_1_*= (8000, 10000).

Evaluation of all particles.

*P_1_= (8000, 10000)**,* the length of the CpG island (*CpG_length_*) is 2,000 (10000-8000) bp. If the number of C (*#C*) is 680, the number of G (*#G*) is 610 and the number of CpG (*#CpG*) is 300. The fitness (*P_1_*) is thus calculated as:

$$Fitness(P_{1})=\frac{680+610}{2000}+\frac{\frac{300}{2000}}{\frac{680}{2000}*\frac{610}{2000}}+\frac{(10000-8000)-200}{2000-200}=0.64+1.44+1=3.08$$

The fitness of *pbest_1_* is 3.08, the fitness of *pbest_2_* is 2.48 and the fitness of *pbest_3_* is 2.34. Since the fitness value of *P_1_* is the highest, *pbest_1_* is now the *gbest*: *gbest = pbest_1._*

Step 5: Update velocity (*V_i_* ) and position (*X_i_*)

At each generation, the position and velocity of every particle is updated according to its own *pbest* and *gbest* by Eq. (6) and Eq. (7).

|  | (6) |
| --- | --- |
| $x_{id}^{new} =x_{id}^{old} +v_{id}^{new}$ | (7) |

where *r_1_* and *r_2_* are random numbers between (0, 1).

Update of *V_1_* and *X_1_*

$v_{1}^{old}$

If the *w* is 1, is (1, 1), *r_1_* is 0.1, *r_2_* is 0.2, and *C_1_* and *C_2_* are 2, respectively.

Since the *Fe* value is higher than the sequence length 10000, the *Fe* value needs to be limited to 10000.

Update of *V_2_* and *X_2_*

$v_{2}^{old}$

If *w* is 1, is (1, 1), *r_1_* is 0.01, *r_2_* is 0.02, and both *C_1_* and *C_2_* are 2.

Update *V_3_* and *X_3_*

$v_{3}^{old}$

If *w* is 1, is (1, 1), *r_1_* is 0.02, *r_2_* is 0.01, *and C_1_* and *C_2_* are 2, respectively.

Step 6: If the stopping criterion is satisfied then the particle results are output.

*P_1_= (8001, 10000),* the length of CpG island (*CpG_length_*) is 1,999 (10000-8001) bp. If the number of C (*#C*) is 680, the number of G (*#G*) is 610 and the number of CpG (*#CpG*) is 300. The fitness (*P_1_*) is thus calculated as:

$$Fitness(P_{1})=\frac{680+610}{1999}+\frac{\frac{300}{1999}}{\frac{681}{1999}*\frac{610}{1999}}+\frac{(10000-8001)-200}{2000-200}=0.64+1.44+0.99=3.07$$

The updated Fitness (*P_1_*) is 3.07. Since the fitness *pbest_1_* is 3.08, *pbest_1_* still remains unchanged.

*P_2_ = (2711, 4240)* the length of CpG island (*CpG_length_*) is 1,529 (4240-2711) bp. If the number of C (*#C*) is 650, the number of G (*#G*) is 600 and the number of CpG (*#G*) is 200. The fitness (*P_1_*) is thus calculated as:

$$Fitness(P_{2})=\frac{650+600}{1529}+\frac{\frac{200}{1529}}{\frac{650}{1529}*\frac{600}{1529}}+\frac{(4351-2851)-200}{2000-200}=0.81+0.78+0.73=2.32$$

The updated fitness (*P_2_*) is 2.32. Since *pbest_2_* is 2.48, *pbest_2_* again remains unchanged.

*P_3_ = (5159, 6277),* the length of CpG island (*CpG_length_*) is 1,118 (6277-5159) bp. If the number of C (*#C*) is 401, the number of G (*#G*) is 391 and the number of CpG (*#CpG*) is 171. The fitness (*P_3_*) is thus calculated as:

$$Fitness(P_{3})=\frac{401+391}{1118}+\frac{\frac{171}{1100}}{\frac{401}{1100}*\frac{391}{1100}}+\frac{(6277-5159)-200}{2000-200}=0.70+1.21+0.51=2.42$$

The updated fitness (*P_3_*) is 2.42, which is better than the original *pbest* fitness. Hence, *pbest*_3_ = (5159, 6277), and its fitness is 2.42.

The final result is

*P_1_= (8000, 10000),* Fitness (*P_1_*) =3.08

*P_2_= (2500, 4000),* Fitness (*P_2_*) =2.48

*P_3_= (5159, 6277),* Fitness (*P_3_*) =2.42

Step 7: RL system


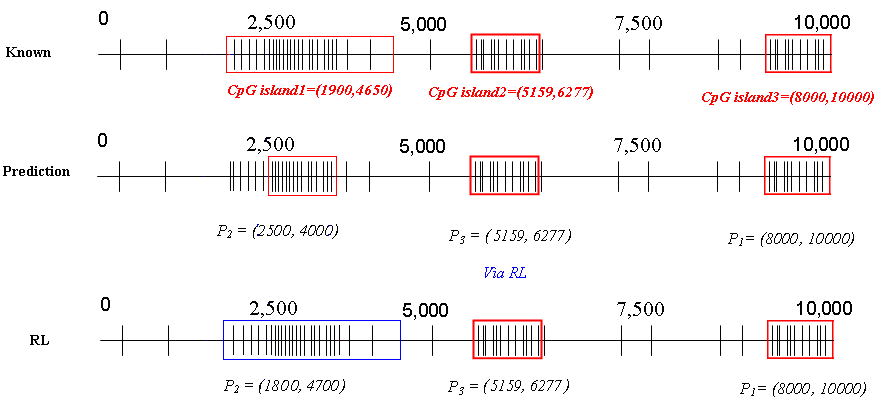


The RL system is applied to extend each CpG island while the prediction still conforms to GGF criteria (not considering the CpG island length). The RL system is then applied to the results of the CPSO algorithm. For example, the CpG island encoded by *P_2_* is extended from (*2500, 4000)* to (*1800, 4700*) by altering the start and end positions.

Example:

The figure above shows that for the known CpG island1 located at 1900 bp~4650 bp, a predicted CpG island is located between 2500 and 4000 before RL is applied (*2500, 4000)*. After the extension, the length of the CpG island (*CpG_length_*) is 2900 (*1800, 4700*) bp and the prediction still remains conform to the GGF criteria (Length≧200, GC content≧0.5 and observed/expected (O/E) ratio≧0.6).

For the original *P_2_ = (2500, 4000),* the length of the CpG island (*CpG_length_*) is 1,500 (4000-2500) bp. If the number of C (#C) is 750, the number of G (#G) is 700 and the number of CpG (#CpG) is 280. The fitness (*P_2_*) is thus calculated as:

$$Fitness(P_{2})=\frac{750+700}{1500}+\frac{\frac{280}{1500}}{\frac{750}{1500}*\frac{700}{1500}}+\frac{(4000-2500)-200}{2000-200}=0.96+0.8+0.72=2.48$$

In the next steps, the RL system is used to extend the CpG island by 10 bp (left shift the start position and right shift the end position by 5bp). *P_2_* is thus extended from (*2500, 4000)* to (2495*, 4005*). If the number of C (*#C*) is 750, the number of G (*#G*) is 702 and the number of CpG (*#CpG*) is 280. The fitness (*P_2_*) is calculated as:

$$Fitness(P_{2})=\frac{750+702}{1510}+\frac{\frac{280}{1510}}{\frac{750}{1510}*\frac{702}{1510}}+\frac{(4005-2495)-200}{2000-200}=0.96+0.80+0.72=2.48$$

The GC content or O/E ratio of each extended CpG island is repeatedly calculated until the GGF criteria are not conformed to anymore (not include CpG island length here). *P_2_* is extended from (*2500, 4000)* to (1800*, 4700*). If the number of C (*#C*) is 750, the number of G (*#G*) is 704 and the number of CpG (*#CpG*) is 280. The fitness (*P_2_*) is calculated as:

$$Fitness(P_{2})=\frac{750+704}{2900}+\frac{\frac{280}{2900}}{\frac{750}{2900}*\frac{704}{2900}}+\frac{(4700-1800)-200}{2000-200}=0.50+1.53+1.5=3.53$$

In the next step both ends of the predicted island are simultaneously extended. If the number of C (*#C*) is 750, the number of G (*#G*) is 704 and the number of CpG (*#CpG*) is 280. *P_2_* is extended from (*1800, 4700)* to (1795*, 4705*). The fitness (*P_2_*) is calculated as:

$$Fitness(P_{2})=\frac{750+704}{2910}+\frac{\frac{280}{2910}}{\frac{750}{2910}*\frac{704}{2910}}+\frac{(4705-1795)-200}{2000-200}=0.49+1.54+1.50=3.53$$

Since the island has a GC content < 0.5, the previous step needs to be rolled back and the start position left-shifted by 10 bp. If the number of C (*#C*) is 750, the number of G (*#G*) is 705 and the number of CpG (*#CpG*) is 280. *P_2_* is extended from (*1800, 4700)* to (1790*, 4700*). The fitness (*P_2_*) is calculated as:

$$Fitness(P_{2})=\frac{750+705}{2910}+\frac{\frac{280}{2910}}{\frac{750}{2910}*\frac{705}{2910}}+\frac{(4700-1790)-200}{2000-200}=0.5+1.54+1.50=3.54$$

Since the extended island has a GC content ≧ 0.5 and conforms to GGF criteria, RL continues to left shift the start position; *P_2_* is extended from (*1790, 4700)* to (1780*, 4700*). If the number of C (*#C*) is 750, the number of G (*#G*) is 705 and the number of CpG (*#CpG*) is 280. The fitness (*P_2_*) is calculated as below:

$$Fitness(P_{2})=\frac{750+705}{2920}+\frac{\frac{280}{2920}}{\frac{750}{2920}*\frac{705}{2920}}+\frac{(4700-1780)-200}{2000-200}=0.49+1.54+1.51=3.54$$

Given that the island has a GC content < 0.5, we again need to roll back the previous step and right shift the end position by 10 bp. If the number of C (*#C*) is 750, the number of G (*#G*) is 705 and the number of CpG (*#CpG*) is 280. *P_2_* is thus extended from (*1790, 4700)* to (1790*, 4710*). The fitness (*P_2_*) is calculated as:

$$Fitness(P_{2})=\frac{750+705}{2920}+\frac{\frac{280}{2920}}{\frac{750}{2920}*\frac{705}{2920}}+\frac{(4710-1790)-200}{2000-200}=0.49+1.54+1.51=3.54$$

Given that the GC content < 0.5, the previous step is undone and RL terminated since the stopping criterion has been reached. Finally, *P_2_* is extended from (*2500, 4000)* to (1790*, 4700*).

*P_1_= (8000, 10000)*

*P_2_ = (1790, 4700)*

*P_3_ = (5159, 6277)*

This result indicates that the CpG islands are located between 1790~4700 bp, 5159~6277 bp and 8000~10000 bp, respectively.
