# Supplementary material for: Particle Swarm Optimization with Reinforcement Learning for the Prediction of CpG Islands in the Human Genome
Source: PLoS One. 2011 Jun 28;6(6):e21036. doi: 10.1371/journal.pone.0021036 (PMC3125183; doi:10.1371/journal.pone.0021036)
Supplement: Text S1 — The pseudo-codes for PSO and CPSO. (DOC) [file pone.0021036.s010.doc]

### Text S1.

### Algorithm design and illustrative example

In this section, we describe the pseudo-code of PSO used to predict CpG islands. The original pseudo-code of the PSO process is shown below.

***Supplementary pseudo-code for*** PSO

| Pseudo-code for PSO |
| --- |
| 1. Begin  2. Randomly initialize particles swarm  3. while(the stopping criterion is not met)  4. Evaluate fitness of particles  5. for n = 1 to number of particles  6. Find *pbest*  7. Find *gbest*  8. for d=1 to number of dimension of particle  9. update the position of particles by Eq. (1)-(2)  10. next d  11. next n  12. update the inertia weight value by Eq.(3)  13. next generation until stopping criterion  14. End |

We describe the pseudo-codes of CPSO-RL. We added a complementary operation after the replacement operation to a standard PSO. The complementary operation changes the search space based on fitness values. It avoids entrapment in a local optimum and improves the balance of global and local search.

(1)

$x_{id}^{new} =x_{id}^{old} +v_{id}^{new}$ (2)

$w=(w_{\max-}w_{\min})\times\frac{move_{\max-}move_{i}}{move_{\max}}+w_{\min}$ (3)

***Supplementary pseudo-code for CPSO***

| Pseudo-code for CPSO |
| --- |
| 1. Begin  2. randomly initialize particles swarm  3. while(the stopping criterion is not met)  4. evaluate fitness of particles  5. for n = 1 to number of particles  6. find *pbest*  7. find *gbest*  8. for d=1 to number of dimension of particle  9. update the position of particles by Eq. (1)-(2)  10. next d  11. next n  12. update the inertia weight value by Eq.(3)  13. if fitness of *gbest* is the same five times then  14. Randomly select a half of particles swarm S  15. Generate new particles C by Eq. (4 ) and replace S  16. end if  17. next generation until stopping criterion  18. End |

In this study, we used reinforcement learning (RL) after the prediction process. Although prediction of CpG islands generally conforms to the GGF criteria, the length of some predicted CpG islands is shorter than the length of known CpG islands. Therefore the result may have a negative impact on the sensitivity (SN). To avoid this, we used RL to combine CpG islands located close to each other into subsets and extend the length of the CpG islands. The pseudo-code for RL is shown below.

***Supplementary pseudo-code for reinforcement learning***

| Pseudo-code for reinforcement learning procedure |
| --- |
| /* set length l was 10 and new_len was CpG island length */  1. Begin  2. length l = 10 ;  3. new_len0 = CpG island length;  4. for ( i = 1 to Number of CpG islands )  5. If (length of CpG islands too shorter )  6. for ( j = 1 to n)  7. new_lenj = length(i) + new_len(j-1) + 1;  8. Estimate new_lenj of CpG islands;  9. If (conform the GGF criteria)  10. record the new_lenj ;  11. update the new_lenj ;  12. Else stop the new_lenj  13. Next j;  14. Next i;  15. End |

***Supplementary Illustrative example***

Example 1 (asymmetric):

$X_{\max}$=2000, $X_{\min}$=200 and dimensions=2 range of solution space.

If selected particles $x_{id}^{selected}$= [500, 1000], then $x_{id}^{complement}$ = [1700, 1200].

$x_{id}^{complement}$= [2200 – 500, 2200 – 1000] = [1700, 1200]

The value 2200 is $X_{\max}$+$X_{\min}$, the value 1700 and 1200 are derived by subtraction $x_{id}^{selected}$acquisition from 2200, respectively.

Example 2 (symmetric):

$X_{\max}$=200, $X_{\min}$=-200 and dimensions=2 range of solution space.

If selected particles $x_{id}^{selected}$= [500, 1000] then $x_{id}^{complement}$ = [-500,-1000].

$x_{id}^{complement}$= [0 – 500, 0 – 1000] = [-500, -1000]

The value 0 is$X_{\max}$+$X_{\min}$, the value -500 and -1000 is 0 are derived by subtraction $x_{id}^{selected}$acquisition, respectively. It can be seen when the $X_{\max}$+$X_{\min}$ is 0, the$x_{id}^{complement}$ is $x_{id}^{selected}$negative number.
