# Supplementary material for: Particle Swarm Optimization with Reinforcement Learning for the Prediction of CpG Islands in the Human Genome
Source: PLoS One. 2011 Jun 28;6(6):e21036. doi: 10.1371/journal.pone.0021036 (PMC3125183; doi:10.1371/journal.pone.0021036)
Supplement: Table S1 — Comparison of different CpG island prediction tools for contig NT_113954.1. (DOC) [file pone.0021036.s011.doc]

**Table S1.** Comparison of different CpG island prediction tools for contig NT_113954.1.

| **Methods** | **Number of CpG islands** | **Start** | **End** | **GC**  **content (%)** | **O/E**  **ratio** | **Length**  **(bp)** | **Total**  **Length (bp)** |
| --- | --- | --- | --- | --- | --- | --- | --- |
| **CpGPlot** | 1  2  3  4 | 50068  58577  63379  90676 | 50488  59076  63738  90893 | 54.76  65.53  68.80  54.84 | 0.737  0.769  0.730  0.738 | 421  500  560  218 1699 | |
| **CpGcluster** | 1  2  3  4  5  6  7 | 2319  50301  58508  58686  62641  63290  90700 | 2700  50405  58622  59101  63118  63683  90915 | 67.80  62.86  67.83  68.27  67.99  73.35  53.24 | 0.584^b^  1.212  0.756  0.784  0.532^b^  0.661  0.860 | 382  105^c^  115^c^  416  478  394  216 2106 | |
| **CpGProd** | 1  2  3  4  5 | 49973  56676  58322  63269  90382 | 50910  57416  59388  64086  90917 | 51.49  52.90  61.48  58.31  50.37 | 0.533^b^  0.604  0.619  0.561^b^  0.589^b^ | 938  741  1067  818  536 4100 | |
| **CpGIS** | 1  2  3  4  5  6  7  8  9  10  11  12  13  14  15  16  17  18  19 | 1765  16553  31025  31557  34421  50093  53162  55001  55855  56571  57343  57832  58533  59612  60646  62858  63402  90719  107454 | 2618  16794  31226  31765  34598  50677  53365  55558  56030  57194  57519  58031  59268  59826  60857  63058  63931  91047  107668 | 44.50^a^  55.00  50.00  50.20  51.10  54.90  56.90  40.10^a^  55.10  39.60^a^  58.80  59.50  63.00  50.20  50.00  63.70  64.30  53.80  50.20 | 0.766  0.602  0.639  0.608  0.602  0.615  0.607  0.796  0.602  0.900  0.603  0.601  0.659  0.608  0.679  0.604  0.639  0.602  0.603 | 854  242  202  209  178^c^  585  204  558  176^c^  624  177^c^  200  736  215  212  201  530  329  215 6647 | |
| **PSO** | 1  2  3  4  5  6  7  8 | 2165  50299  54905  56378  57772  58271  63287  90346 | 2531  50500  55140  57363  58016  59466  64245  90889 | 60.38  60.20  53.62  54.31  60.66  58.91  50.10  50.46 | 0.603  0.886  0.618  0.600  0.629  0.614  0.632  0.611 | 367  202  236  986  245  1196  959  544 4735 | |
| **PSORL** | 1  2  3  4  5  6  7  8 | 2155  49919  54905  56378  57762  58121  63257  90336 | 2531  50510  55160  57363  58026  59476  64255  90889 | 59.31  55.49  52.94  54.31  59.09  58.82  50.00  50.09 | 0.609  0.601  0.600  0.600  0.620  0.603  0.609  0.608 | 377  592  256  986  265  1356  999  554 5385 | |
| **CPSO** | 1  2  3  4  5  6  7  8  9  10  11 | 1692  2204  16496  50110  53068  54918  56514  58165  62679  63319  90701 | 1950  2403  16714  50498  53370  55190  57382  59400  62953  54158  90893 | 54.26  59.69  56.88 55.52  56.62  51.84  54.26  59.68  64.96  51.37  54.17 | 0.654  0.688  0.652  61.56  0.613  0.633  0.615  0.616  0.649  0.650  0.786 | 259  200  219  389  303  273  869  1236  275  840  201 5064 | |
| **CPSORL** | 1  2  3  4  5  6  7  8  9  10  11 | 1652  2152  16486  49918  53068  54898  56483  58136  62669  63259  90621 | 1970  2568  16714  50905  53370  55190  57375  59400  62953  64226  90893 | 50.00  60.82  55.70  50.69  56.62  53.42  54.26  59.97  65.14  50.46  56.25 | 0.621  0.604  0.624  0.608  0.614  0.601  0.613  0.603  0.620  0.601  0.618 | 319  417  229  988  303  293  893  1265  285  968  273 6233 | |

^a^The contig violates the GGF criteria because the GC content is less than 50%.

^b^The contig violates the GGF criteria because the island length is less than 200 bp.

^c^The contig violates the GGF criteria because the O/E ratio is less than 0.6.
